# Genome-wide analysis reveals regulatory mechanisms and expression patterns of *TGA* genes in peanut under abiotic stress and hormone treatments

**DOI:** 10.3389/fpls.2023.1269200

**Published:** 2023-11-21

**Authors:** Chao Zhong, Yu Liu, Zhao Li, Xiaoguang Wang, Chunji Jiang, Xinhua Zhao, Shuli Kang, Xibo Liu, Shuli Zhao, Jing Wang, He Zhang, Yuning Huang, Haiqiu Yu, Renfeng Xue

**Affiliations:** ^1^ College of Agronomy, Shenyang Agricultural University, Shenyang, China; ^2^ Crop Research Institute, Liaoning Academy of Agricultural Sciences, Shenyang, China; ^3^ Liaoning Provincial Key Laboratory of Miscellaneous Grain Germplasm Innovation and Genetic Breeding, Liaoning Academy of Agricultural Sciences, Shenyang, China

**Keywords:** *TGA* genes, peanut (*Arachis hypogaea*), abiotic stress, hormone signaling, transcription factors

## Abstract

**Introduction:**

The *TGA* transcription factors, plays a crucial role in regulating gene expression. In cultivated peanut (*Arachis hypogaea*), which faces abiotic stress challenges, understanding the role of *TGAs* is important.

**Methods:**

In this study, we conducted a comprehensive in analysis of the *TGA* gene family in peanut to elucidate their regulatory mechanisms and expression patterns under abiotic stress and hormone treatments. Furthermore, functional studies on the representative *AhTGA* gene in peanut cultivars were conducted using transgenic *Arabidopsis* and soybean hair roots.

**Results:**

The genome-wide analysis revealed that a total of 20 *AhTGA* genes were identified and classified into five subfamilies. Collinearity analysis revealed that *AhTGA* genes lack tandem duplication, and their amplification in the cultivated peanut genome primarily relies on the whole-genome duplication of the diploid wild peanut to form tetraploid cultivated peanut, as well as segment duplication between the A and B subgenomes. Promoter and Protein-protein interaction analysis identified a wide range of cis-acting elements and potential interacting proteins associated with growth and development, hormones, and stress responses. Expression patterns of *AhTGA* genes in different tissues, under abiotic stress conditions for low temperature and drought, and in response to hormonal stimuli revealed that seven *AhTGA* genes from groups I (*AhTGA04*, *AhTGA14* and *AhTGA20*) and II (*AhTGA07*, *AhTGA11*, *AhTGA16* and *AhTGA18*) are involved in the response to abiotic stress and hormonal stimuli. The hormone treatment results indicate that these *AhTGA* genes primarily respond to the regulation of jasmonic acid and salicylic acid. Overexpressing *AhTGA11* in *Arabidopsis* enhances resistance to cold and drought stress by increasing antioxidant activities and altering endogenous hormone levels, particularly ABA, SA and JA.

**Discussion:**

The *AhTGA* genes plays a crucial role in hormone regulation and stress response during peanut growth and development. The findings provide insights into peanut's abiotic stress tolerance mechanisms and pave the way for future functional studies.

## Introduction

Transcription factors (*TFs*) play a critical role in mediating the response of plants to changes in their external environment (Javed et al., 2020; Hrmova and Hussain, 2021; Song et al., 2022). By binding to specific DNA sequences, transcription factors can activate or repress the expression of genes (Latchman, 2001). They can also interact with other signaling molecules and proteins to modulate the expression of target genes, enabling plants to quickly and effectively respond to environmental challenges such as drought, heat, cold, or pathogen attack (Fraire-Velázquez et al., 2011; Nakashima et al., 2014; Innes, 2018; Srivastava et al., 2023). The ability of transcription factors to integrate diverse signals and coordinate gene expression makes them essential components of the complex regulatory networks that control plant responses to environmental stress (Valliyodan and Nguyen, 2006; Borrill et al., 2019; Kidokoro et al., 2022). Understanding the mechanisms by which transcription factors regulate gene expression in response to environmental cues is crucial for developing strategies to enhance plant resilience and improve agricultural productivity.

The basic leucine zipper (*bZIP*) gene family, one of the largest transcription factor families in plants, is categorized into ten groups (A, B, C, D, E, F, G, H, I, and S), along with two additional groups, J and K. This classification is based on the similarity in the basic region and additional conserved motifs (Jakoby et al., 2002; Nijhawan et al., 2008; Wang et al., 2015). TGA (TGACG motif-binding factor) transcription factors are part of group D, which recognizes as-1-type *cis-*elements found in the promoter regions of target genes (Gatz, 2013; Liu et al., 2022b). The bZIP domain’s primary structure in TGA proteins is highly conserved and includes an invariant motif N-x7R/K-x9-L-x6-L-x6-L in the N-terminus. In the C-terminus, the motif Yx2RL[RQ]ALSS[LS]W represents the signature domain of group D (Tomaz et al., 2022). Since the first *TGA* gene *TGA1a* was identified in tobacco, this gene family has been isolated and identified in various species, including *Arabidopsis*, rice and soybean (Johnson et al., 2003; Fitzgerald et al., 2005; Li et al., 2022). In the *Arabidopsis* genome, there are a total of 10 *TGA* transcription factors that can be divided into five groups based on their sequence similarity (Gatz, 2013). Group I comprise *TGA1* and *TGA4*, which are the most comparable to tobacco *TGA1a* (Budimir et al., 2021; Kim et al., 2022). Group II consists of *TGA2*, *TGA5*, and *TGA6*, which are closely related and have functional overlap with Group I (Zhang et al., 2003; Fonseca et al., 2022). Group III includes *TGA3* and *TGA7*, Group IV includes *TGA9* and *TGA10*, while Group V has only one member, *PERIANTHIA* (*PAN*) (Murmu et al., 2010; Noshi et al., 2016). Numerous studies have been conducted to understand the role of *TGA* genes, *TGA1*-*TGA7* have been widely demonstrated to enhance plant resistance to biotic and abiotic stresses, while *TGA9*, *TGA10* and *PERIANTHIA* (*PAN*) were proved to be involved in the development of plant floral organs (Murmu et al., 2010; Li et al., 2019b; Budimir et al., 2021; Liu et al., 2022a). The *TGA* gene participates in the regulation of multiple hormone signaling pathways, including salicylic acid, jasmonic acid, ethylene, and cytokinin, by interacting with key regulatory factors such as *NPR1*, *GRX480*, *ERF72*, *SCL14* and *BIN2* in plants (Johnson et al., 2003; Fode et al., 2008; Choi et al., 2010; Zander et al., 2010; Jin et al., 2018; Kim et al., 2022).

Cultivated peanuts (*Arachis hypogaea*), a vital cash crop cultivated in tropical and subtropical regions globally, serve as a primary source of both oil and protein on a global scale. Peanuts hold considerable economic and nutritional importance worldwide and play a crucial role in the agricultural and food industries of numerous countries (Syed et al., 2021). Nevertheless, a significant portion of the world’s peanut cultivation takes place on suboptimal soils with limited resources in many developing nations, leading to a substantial disparity between demand and supply (Zhang et al., 2023). In the face of climate change, various abiotic factors such as drought and temperature fluctuations impose constraints on both the quality and productivity of peanut crops (Dwivedi et al., 2013; Puppala et al., 2023). Hence, there is an urgent necessity to pinpoint pivotal genes capable of conferring tolerance to abiotic stress, which can then be harnessed in biotechnological initiatives aimed at developing enhanced peanut varieties (Baillo et al., 2019; Dormatey et al., 2020). Cultivated peanuts are natural allotetraploids originating from the hybridization of two diploid species, *A. duranensis* and *A. ipaensi*s. The complete sequencing of the genomes of A. duranensis, A. ipaensis, and *A. hypogaea* has opened up new avenues for genomic research focusing on functional genes within the peanut (Bertioli et al., 2016; Bertioli et al., 2019). Presently, recent studies have shed light on the potential roles of *TGA* transcription factors in responding to abiotic stress. However, there have been no reports regarding their role in cultivated peanuts (Li et al., 2019a). In this study, we focused on *TGA* genes from *A. hypogaea* genome.

Our analysis included their phylogenetic relationships, conserved domains, gene structures, expansion patterns, *cis-*regulatory elements, protein-protein interactions, and expression profiles in various tissues and under different abiotic stresses. Furthermore, we conducted initial functional validation of key *TGA* genes in *Arabidopsis* and soybean. Our findings provide a comprehensive understanding of *TGA* genes in peanut, and offer a foundation for future functional studies to investigate their roles in regulating peanut’s tolerance to abiotic stress.

## Materials and methods

### Plant materials and treatment

The peanut varieties Nonghua5 (NH5, drought-tolerant and cold-tolerant genotype) and Fuhua18 (FH18, sensitive genotype) were selected as the plant material (Jiang et al., 2020; Zhang et al., 2020; Ren et al., 2022). The young peanut plant seedlings were grown in vermiculite with a light cycle of 16 hours of light (28°C) followed by 8 hours of dark (25°C). After 20 days from sowing, the seedlings were used to investigate their response to various hormones and abiotic stresses.

For cold stress treatment, the temperature in the climate chamber was reduced to 6°C while maintaining other growth conditions. For drought stress treatment, the seedlings were first allowed to recover in hydroponic cultures for 3 days before being subjected to the stress treatment. Subsequently, the seedlings were incubated in a 20% (w/v) solution of polyethylene glycol (PEG-6000). For various abiotic stress treatments, the second leaves were harvested at 0, 6, 12, 24, and 48 hours post-treatment, with three independent replicates. These collected leaves were rapidly frozen in liquid nitrogen and stored at -80°C. To investigate responses to different hormones, including methyl salicylate (MeSA) (0.1 mmol/L), methyl jasmonate (MeJA) (0.1 mmol/L), gibberellin (GA) (0.1 mmol/L), and abscisic acid (ABA) (0.1 mmol/L), they were applied as sprays onto the leaves of cultivated peanut seedlings, while sterile water was utilized as a control. Leaf samples were then collected at 0, 6, 12, 24, and 48 hours after treatment and stored at -80°C for RNA extraction. The phenotypic changes of the plants were observed on the fourth day after treatment.

### Identification of *TGA* family members in cultivated and wild peanut

To identify the members of the *TGA* gene family in cultivated and wild peanut species, we obtained ten reported protein sequences of *Arabidopsis* TGA members from the *Arabidopsis* information resource TAIR (https://www.Arabidopsis.org/). These sequences served as queries to conduct searches for potential *TGA* genes within the genomes of cultivated peanut and two wild peanut species, utilizing the peanut genome database accessible at https://peanutbase.org/ using BLAST (E-value ≤ 10^-5^). The identification of *TGA* genes was carried out by predicting protein domains using the Pfam website (https://pfam.xfam.org/) and the SMART website (https://smart.embl.de/), followed by the removal of redundant sequences. Finally, the members of the *TGA* gene family were determined. To further analyze the characteristics of the peanut *TGA* gene family, we predicted their molecular weight, isoelectric point, and other physicochemical properties using the online software ExPASy (https://www.expasy.org/). Additionally, the subcellular localization of the peanut *TGA* gene family members was predicted using the online software WoLF PSORT (https://www.genscript.com/wolf-psort.html).

### Phylogenetic, conserved motif and gene structure analysis of *AhTGA* genes

Sequence alignment was performed using ClustalW with default parameters (Larkin et al., 2007) to align all candidate *AhTGA* amino acid sequences with *TGA* family members from other species, including *Arabidopsis thaliana*, *Phaseolus vulgaris*, *Glycine Max*, *Cicer arietinum*, *Oryza Sativa*, *Zea mays*, *Sorghum bicolor*, *Vitis vinifera*, and *Medicago truncatula*. For phylogenetic analysis, MEGA 11.0 software was employed, applying the neighbor-joining (NJ) method with the Poisson model, pairwise deletion, and 1,000 bootstrap replications (Tamura et al., 2021). The resulting phylogenetic tree was visualized using the Evolview v2 webserver (He et al., 2016). To analyze the protein structure domains of TGA family proteins, we utilized the SMART online tool (https://smart.embl.de/), and a functional domain diagram was created using the Conserved Domain Database (CDD) from NCBI (https://www.ncbi.nlm.nih.gov/cdd/). The conserved amino acid motifs within the candidate *AhTGA* gene sequences were predicted using the MEME Suite 5.4.1 (https://meme-suite.org/meme/doc/meme.html) with default parameters. The resulting conserved motifs were visualized, and the gene structure diagrams were generated using the Gene Structure View program in TBtools (Chen et al., 2020).

### Chromosomal locations, gene duplications, and synteny analysis

Chromosome length and position information of the 40 *AhTGA* members were extracted from the peanut genome and annotation files. Gene visualization was performed using the MG2C online software (http://mg2c.iask.in/mg2c_v2.1/), while gene duplication events were analyzed using MCScanX with default parameters. To demonstrate the collinearity of the *TGA* gene family, we employed the Advanced Circos function in TBtools. Additionally, the Multiple Synteny Plot was used to illustrate the synteny relationships between *A. hypogaea* and nine other species. The Ka (nonsynonymous substitution rate) and Ks (synonymous substitution rate) were examined using TBtools software, and the calculation of selection pressure was based on the Ka/Ks ratio, as detailed in the work by Wang et al. (2010).

### Protein-protein and microRNAs-AhTGAs interaction network

The interaction relationships between AhTGAs and other proteins were analyzed using the STRING database (https://string-db.org), with a confidence score threshold set at > 0.4. *Arabidopsis thaliana* was used as the query organism. the visualization of the predicted protein-protein interaction (PPI) network was accomplished using Cytoscape 3.9.1 software (Shannon et al., 2003). To predict miRNA-target relationships for *AhTGA*, the psRNA Target Server (http://plantgrn.noble.org/psRNATarget/) was used, with an expected value set to the default value of 5, using the CDS sequence of *AhTGAs* as the candidate target. The predicted miRNA and their corresponding target genes were displayed using Cytoscape 3.9.1 software.

### Conserved *cis-*element analysis in promoters

The promoter region was defined by extracting the upstream 2000 bp sequences of the *TGA* genes from the peanut genome database. To analyze and quantify the presence of *cis-*acting elements associated with growth, development, hormones, and stress response in this promoter region, we utilized the PlantCARE database (http://bioinformatics.psb.ugent.be/webtools/plantcare/html/).

### Transcriptome-based expression pattern of *AhTGAs*

The orthologous genes for the 20 *AhTGA* genes were obtained through BLAST and the gene’s chromosomal locations using http://peanutgr.fafu.edu.cn/index.php (Zhuang et al., 2019). Furthermore, the FPKM values of these genes under hormone, low-temperature, and drought treatments were obtained. Transcriptome data, provided by Clevenger et al. (2016), of 22 different tissues in peanut were obtained from the Phytozome 13 database. The FPKM values of *TGA* genes in cultivated peanut tissues were converted to log_2_^FPKM^ and standardized. Heatmap clustering was performed using the HeatMap function in TBtools software.

### Quantitative RT−PCR validation

RNA extraction was performed on leaves and roots obtained using the Plant Total RNA Extraction Kit from Tiangen Biotech, Beijing, China. The resulting RNA was used to create cDNA using the PrimeScript™ RT Kit from TaKaRa, Japan, following the manufacturer’s instructions. Primers to identify *AhTGA* genes with differential expression were obtained from PrimerBlast (https://www.ncbi.nlm.nih.gov/tools/primer-blast/), and *Actin11* was used as the internal control. The gene expression analysis was carried out using the SYBR Premix Ex TaqII kit (TliRNaseH Plus) from TaKaRa, Japan, and fluorescence quantitative reactions were detected using ABI7500 from Applied Biosystems, United States. The relative expression analysis was calculated using the 2^−ΔΔCT^ approach (Livak and Schmittgen, 2001).

### The *AhTGA11* function analysis under chilling and drought stress conditions in *Arabidopsis* Plants

Gene-specific primers were designed using Primer Premier 6.0 to amplify the cDNA sequence of *AhTGA11* in FH18. PCR amplification of *AhTGA11*’s coding sequence was conducted using the TransTaq DNA Polymerase High Fidelity Amplification Kit (Transgen Biotech). The resulting PCR products were visualized on a 1% agarose gel, purified using a DNA purification recovery kit, and then inserted into the pBWA (V) BS cloning vector driven by the CaMV35S promoter. The ligated DNA was introduced into *Escherichia coli Top10* competent cells, and positive clones were selected and confirmed by sequencing. The recombinant plasmid pBWA (V) BS- AhTGA11 was subsequently transferred into *Agrobacterium tumefaciens* EHA105, which was used to transform wild-type (WT) *Arabidopsis* plants via the floral dip method. After screening with antibiotics and verifying the transgenic seedlings through PCR, homozygous transgenic lines were successfully obtained in the T_2_ generation. Subsequently, homozygous T_3_ progeny were examined and selected for further experimental procedures. The Col-0 seeds and empty half MS medium as control. 12 d old seedlings were transferred to new plates containing no or 8% (w/v) PEG for 5 days. The plant phenotypes were measured, and leaf and root tissues were collected for the measurement of relevant physiological and hormone indicators. For cold treatment, the 18 d old seedlings were treated at 4°C for 5 days, and the phenotypes, physiology, and hormones analyzed.

### Generation of soybean hairy roots using *Agrobacterium Rhizogenes* transformation

The *AhTGA11* gene was amplified and cloned into the pCAMBIA3301 vector under the control of the CaMV35S promoter to generate the pCAMBIA3301-AhTGA11 overexpression vector. Transgenic hairy roots were induced following the method described by Kereszt et al. (2007). The pCAMBIA3301-AhTGA11 vector was transformed into *Agrobacterium* strain K599, and soybean cultivar Williams seedlings at 7 days old were inoculated with the transformed K599 cells for treatment, while soybean plants induced with the K599 strain carrying pCAMBIA3301 were used as controls. The induction of hairy roots was carried out under 90% humidity conditions, and when the hairy roots reached a length of 3-4 cm, the plants were placed in a solution of 8% (w/v) PEG to promote their stable growth. After 4 days of treatment, phenotypic observations and physiological indicators in the roots and leaves were measured.

### Physiological and hormone measurements

Physiological indicators and hormone levels were determined in both overexpressed *Arabidopsis* plants and soybean transgenic plants containing roots. The H_2_O_2_ content was measured following the method described by Sagisaka (1976), malondialdehyde (MDA) content and superoxide dismutase (SOD) activity were determined following the method of Zhang H. et al., 2019, and peroxidase (POD) activity was measured according to the method of Do et al. (2003). Quantitative analysis of hormones in plant samples was performed using liquid chromatography-tandem mass spectrometry (LC-MS/MS) technology, following the procedures outlined in studies (Pan et al., 2010; Cai et al., 2014; Li et al., 2016).

### Statistical analysis

The analyses of statistically significant data were processed by a one-way analysis of variance (ANOVA). In the case of multiple comparisons between different groups, Dunnett’s multiple comparison test or the Student’s *t*-test method was used. All data were analyzed using R language (version 4.3.0), and all statistical analyses in this study were conducted using the respective R packages. All the values were calculated as the means ± standard deviation (SD). Asterisks indicate significant differences. *P < 0.05. * *P < 0.01. Error bars represent the standard deviation from triplicate values.

## Results

### Genome-wide identification of *TGA* genes in cultivated and wild peanut

We conducted a comprehensive search in the peanut genome database using ten *Arabidopsis* TGA protein sequences as references, resulting in the identification of 40 candidate genes (**Supplementary Table 1**). Subsequently, structural domain analysis was performed on all sequences, revealing that the gene sequences of cultivated peanut and the two wild peanut species, *A. duranensis* and *A. ipaensis*, all contained intact bZIP and DOG1 domains (**Supplementary Figure 1**). Specifically, the cultivated peanut genome encompassed 20 *AhTGA* genes, designated as *AhTGA01*-*AhTGA20* based on their respective chromosomal locations (**Table 1**). Similarly, the wild peanut genomes harbored 20 *TGA* genes, denoted as *AdTGA01*-*AdTGA09* and *AiTGA01*-*AiTGA11*, distributed across eight chromosomes. We further analyzed the candidate TGA protein sequences (pI) (**Table 1**). The coding lengths of the 20 AhTGA protein sequences ranged from 331 to 531 amino acids. The predicted MW and pI of AhTGA proteins varied between 37.02 (AhTGA09 and AhTGA18) and 59.17 (AhTGA13) kDa, and 5.84 (AhTGA05) and 8.34 (AhTGA04 and AhTGA14), respectively. In the wild peanut species, the coding lengths of the 20 TGA protein sequences ranged from 331 to 538 amino acids in *A. duranensis* and from 331 to 550 amino acids in *A. ipaensis*. The expected MW of these TGA proteins ranged from 37.02 kDa (AdTGA07) to 59.63 kDa (AdTGA05) in *A. duranensis*, and from 37.02 kDa (AiTGA08) to 61.13 kDa (AiTGA06) in *A. ipaensis*. Moreover, the pI values ranged from 5.84 (AdTGA04) to 8.63 (AdTGA07) in *A. duranensis*, and from 5.97 (AiTGA04) to 8.63 (AiTGA08) in *A. ipaensis*. Subcellular localization predictions indicated that *AhTGA*, *AdTGA*, and *AiTGA* genes were primarily localized in the nucleus. Notably, the *AhTGA* genes in cultivated peanut and the wild diploid peanut species exhibited similar lengths and physical properties.

**Table 1:** The genomic and biochemical information for TGA genes identified in cultivated peanut and two wild.

GENE NAME	Locus ID	Chromosomal Location	DNA(bp)	mRNA(bp)	Protein(aa)	MWb(kDa)	pI	Subcellular Location
*AhTGA01*	*ArahyR4ID1P*	Arahy.02:74466805-74478269	11464	1892	503	56.63	6.69	nucl: 13
*AhTGA02*	*ArahyLTZY89*	Arahy.03:245406-250717	5311	2244	467	51.96	6.32	nucl: 14
*AhTGA03*	*ArahyS1DMKE*	Arahy.03:279111-284423	5312	2244	467	51.96	6.32	nucl: 14
*AhTGA04*	*ArahyRIV6ZB*	Arahy.03:123676732-123681028	4296	1944	368	41.63	8.34	nucl: 11, chlo: 3
*AhTGA05*	*Arahy0GP62G*	Arahy.04:47164073-47171787	7714	1421	388	43.62	5.84	nucl: 11, cyto: 1, pero: 1
*AhTGA06*	*Arahy661MCQ*	Arahy.05:109518217-109525080	6863	1584	459	51.24	6.21	nucl: 10, chlo: 4
*AhTGA07*	*ArahyKZ9VWQ*	Arahy.06:10302739-10309738	6999	2211	450	49.51	7	nucl: 14
*AhTGA08*	*ArahyJ1CFJJ*	Arahy.08:30570146-30577442	7296	1932	487	53.62	7.38	nucl: 13
*AhTGA09*	*ArahyRKP385*	Arahy.08:43602879-43611829	8950	1998	331	37.02	8.63	nucl: 12, nucl_plas: 8
*AhTGA10*	*ArahyW15JJQ*	Arahy.09:113914413-113918157	3744	2553	456	50.92	6.31	nucl: 11, chlo: 3
*AhTGA11*	*ArahyP1JBJN*	Arahy.10:115248749-115254745	5996	2141	463	51.15	6.53	nucl: 14
*AhTGA12*	*Arahy5F9GBV*	Arahy.12:88825865-88838186	12321	1899	504	56.71	6.56	nucl: 13
*AhTGA13*	*ArahyEH3L7F*	Arahy.13:2283773-2288910	5137	2213	531	59.17	6.49	nucl: 14
*AhTGA14*	*Arahy6K442L*	Arahy.13:126982024-126986332	4308	1944	368	41.59	8.34	nucl: 10, chlo: 4
*AhTGA15*	*ArahyH6GQLR*	Arahy.15:121748615-121755797	7182	1694	469	52.08	5.97	nucl: 10, chlo: 4
*AhTGA16*	*ArahyPHPT4F*	Arahy.16:9377082-9384326	7244	3502	431	47.17	6.21	nucl: 14
*AhTGA17*	*Arahy980X4K*	Arahy.18:6346988-6353680	6692	2011	492	54.38	8.27	nucl: 12, cyto: 1
*AhTGA18*	*ArahySERN02*	Arahy.18:122380117-122388818	8701	2013	331	37.02	8.63	nucl: 12, nucl_plas: 8
*AhTGA19*	*ArahyK04A4E*	Arahy.19:154421962-154425784	3822	2494	456	50.97	6.34	nucl: 10.5, cyto_nucl: 6, chlo: 3
*AhTGA20*	*ArahyAAI19J*	Arahy.20:23833950-23839123	5173	1848	362	41.03	6.63	nucl: 6, chlo: 5, mito: 3
*AdTGA01*	*AraduX4N5C*	Aradu.A02:67538591-67544697	6106	6106	416	46.84	5.98	nucl: 14
*AdTGA02*	*AraduU5DG8*	Aradu.A03:114621519-114625756	4237	4237	368	41.63	8.34	nucl: 11, chlo: 3
*AdTGA03*	*AraduM9L15*	Aradu.A04:72882430-72890856	8426	8426	373	42	5.97	nucl: 11, cyto: 2
*AdTGA04*	*AraduC716W*	Aradu.A05:103557314-103564234	6920	6920	476	53	5.81	nucl: 14
*AdTGA05*	*AraduBQ4IU*	Aradu.A06:9768086-9774583	6497	6497	538	59.63	8.32	chlo: 7, nucl: 6
*AdTGA06*	*AraduI6GFF*	Aradu.A08:28342298-28348900	6602	6602	492	54.28	7.1	nucl: 14
*AdTGA07*	*AraduGUH8G*	Aradu.A08:41608257-41616928	8671	8671	331	37.02	8.63	nucl: 12, nucl_plas: 8
*AdTGA08*	*Aradu1JQ3A*	Aradu.A09:114398072-114400850	2778	2778	485	54.25	6.37	chlo: 6, nucl: 5, mito: 2
*AdTGA09*	*AraduCM60U*	Aradu.A10:15794805-15799916	5111	5111	414	47.24	6.53	nucl: 11, chlo: 1, plas: 1
*AiTGA01*	*AraipI0X2E*	Araip.B02:78769485-78775072	5587	5587	397	44.45	6.17	nucl: 13
*AiTGA02*	*AraipHE4VX*	Araip.B03:2236977-2240401	3424	3424	448	50.14	8.41	nucl: 13
*AiTGA03*	*AraipW8KP6*	Araip.B03:116847342-116851500	4158	4158	368	41.59	8.34	nucl: 10, chlo: 4
*AiTGA04*	*AraipNZ32C*	Araip.B04:65308191-65317689	9498	9498	373	42.05	5.97	nucl: 11, cyto: 2
*AiTGA05*	*AraipX0N4G*	Araip.B05:112439034-112446285	7251	7251	479	53.05	6.06	nucl: 12, chlo: 2
*AiTGA06*	*AraipW3SNY*	Araip.B06:6055755-6061740	5985	5985	550	61.13	7.64	chlo: 9, nucl: 4
*AiTGA07*	*Araip1FG0Z*	Araip.B08:5553954-5560404	6450	6450	467	51.27	6.92	nucl: 14
*AiTGA08*	*AraipD5QVM*	Araip.B08:117483523-117491929	8406	8406	331	37.02	8.63	nucl: 12, nucl_plas: 8
*AiTGA09*	*AraipUIF5W*	Araip.B09:143024347-143027150	2803	2803	456	50.97	6.34	nucl: 10.5, cyto_nucl: 6, chlo: 3
*AiTGA10*	*AraipS8WR7*	Araip.B10:22665260-22670590	5330	5330	356	40.35	6.63	nucl: 6, chlo: 5, mito: 3
*AiTGA11*	*Araip7QE9U*	Araip.B10:134367929-134373817	5888	5888	433	48.1	6.04	nucl: 13

### Phylogenetic analysis of the *TGA* genes in cultivated peanut

**Figure 1:**
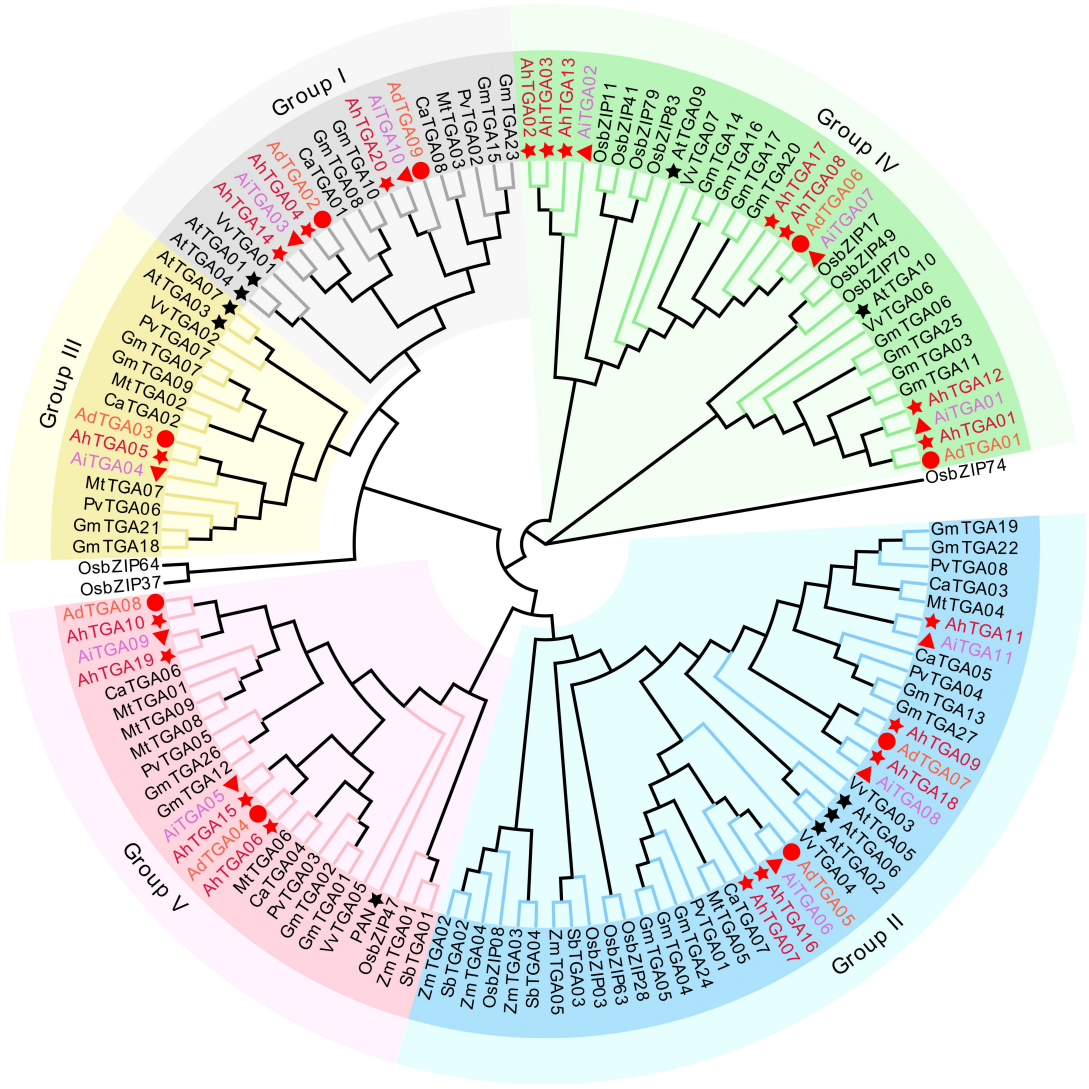
A neighbor-joining phylogenetic tree was created using 133 TGA proteins from various plant species, including *Arabidopsi*s (At), chickpea (Ca), alfalfa (Mt), common beans (Pv), soybean (Gm), grapes (Vv), rice (Os), sorghum (Sb), maize (Zm), and three *Arachis* species. The TGA proteins were categorized into five clades, distinguished by distinct background colors. TGA proteins found in cultivated and wild peanuts are denoted by red stars, circles, and triangles, respectively.

The phylogenetic tree was constructed for peanut, *Arabidopsis*, common bean, soybean, grape, alfalfa, chickpea, rice, maize, and sorghum (**Supplementary Table 1**). Based on the classification of 133 TGA proteins with reference to *Arabidopsis* TGA proteins, the TGA proteins were divided into five groups (**Figure 1**), namely Group I-Group V, each of which contained 41, 25, 15, 18, and 31 members, respectively. AhTGA04, AhTGA14, and AhTGA20 belonged to branch I, AhTGA07, AhTGA09, AhTGA11, AhTGA16, and AhTGA18 belonged to branch II, AhTGA05 belonged to branch III, AhTGA01, AhTGA02, AhTGA03, AhTGA08, AhTGA12, AhTGA13, and AhTGA17 belonged to branch IV, and AhTGA06, AhTGA10, AhTGA15, and AhTGA19 belonged to branch V. Phylogenetic analysis showed that peanut TGA proteins had high similarity and genetic distance with the protein sequences of other leguminous plants, indicating that they may have similar functions.

### Gene structure and conserved motifs analysis

**Figure 2:**
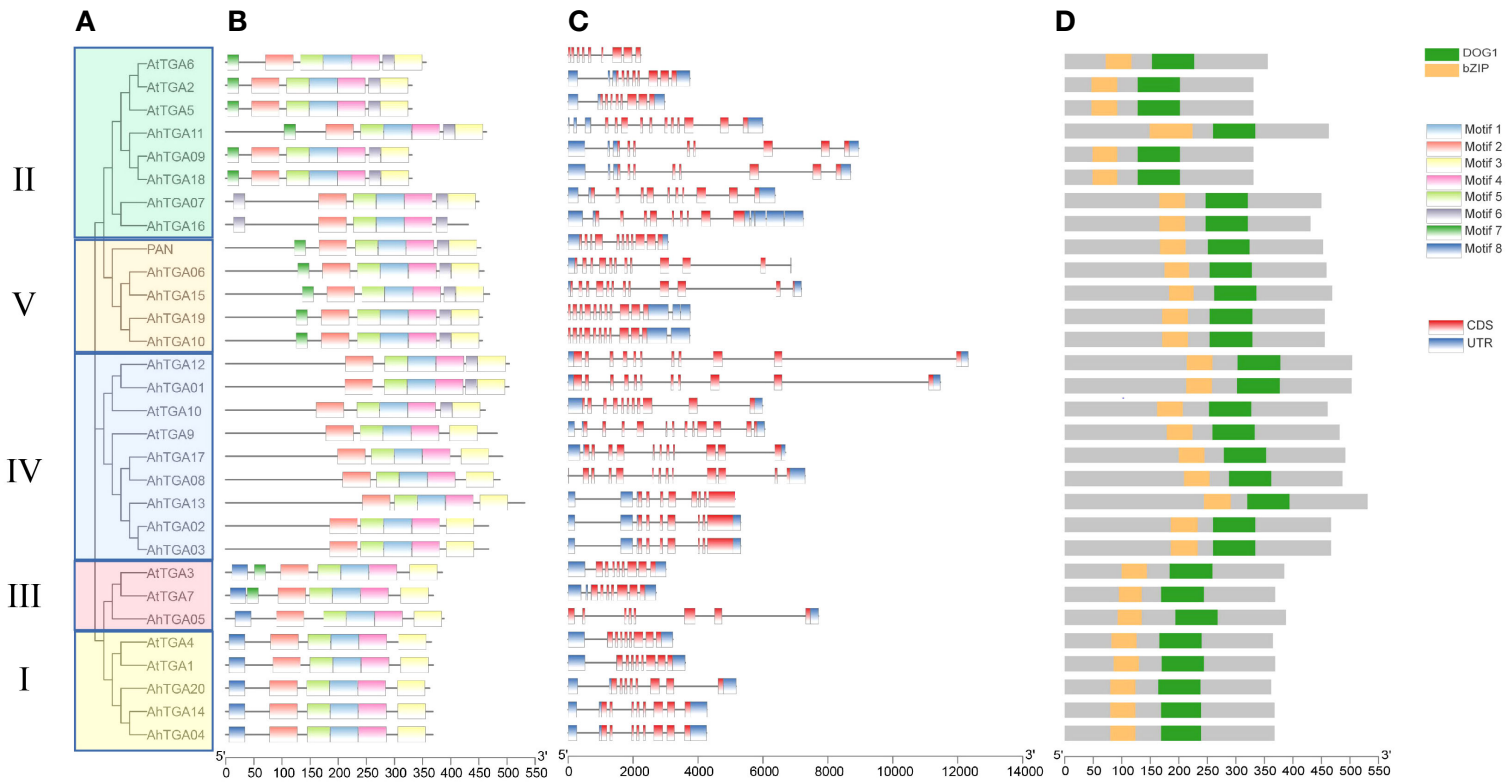
Phylogenetic analysis, conserved motifs, gene structures, and conserved domains of *TGA* genes in both cultivated peanut and Arabidopsis were examined. **(A)** A phylogenetic tree was generated using the Neighbor-Joining (NJ) method, illustrating the relationships among TGA protein sequences in cultivated peanut and *Arabidopsis*. **(B)** Conserved motifs within AhTGAs were identified using MEME, with different colors representing distinct motifs. **(C)** The structural characteristics of twenty *AhTGA* genes were analyzed. **(D)** A comparison of conserved domains between AtTGAs and AhTGAs was conducted, with the ruler at the bottom indicating sequence lengths.

In cultivated peanut proteins, up to 8 motifs were identified, with a total length of 21-50 amino acids (**Figures 2A, B**; **Supplementary Figure 2**; **Supplementary Table 2**). The number of motifs in TGA proteins ranged from 5 to 7, all of which contained the 5 conserved motifs, Motif 1 to Motif 5. There were differences in motif distribution among different branches, with Motif 6 found only in branches II, IV, and V; Motif 7 only in branches II, and III and V; and Motif 8 only in groups I and III. Differences in motif distribution may lead to changes in *TGA* gene structure and function (**Figure 2B**). To analyze the relationship between genome evolution and functional differentiation, the gene structure of *AhTGAs* was further analyzed. The number of exons in the *AhTGA* gene family ranged from 9 to 16. *TGA* genes with close evolutionary relationships not only had the same number of exons, but also had similar structures (**Figure 2C**). According to conserved domain analysis, both bZIP and DOG1 domains were found in each AhTGA protein, and these domains were located in similar positions in different sequences (**Figure 2D**). These results suggest that *AhTGA* genes have conserved structural domains in gene structure, but specific sequence structures exist in *TGA* genes in different clades.

### Chromosomal locations, gene duplication and syntenic analysis of *AhTGAs*

**Figure 3:**
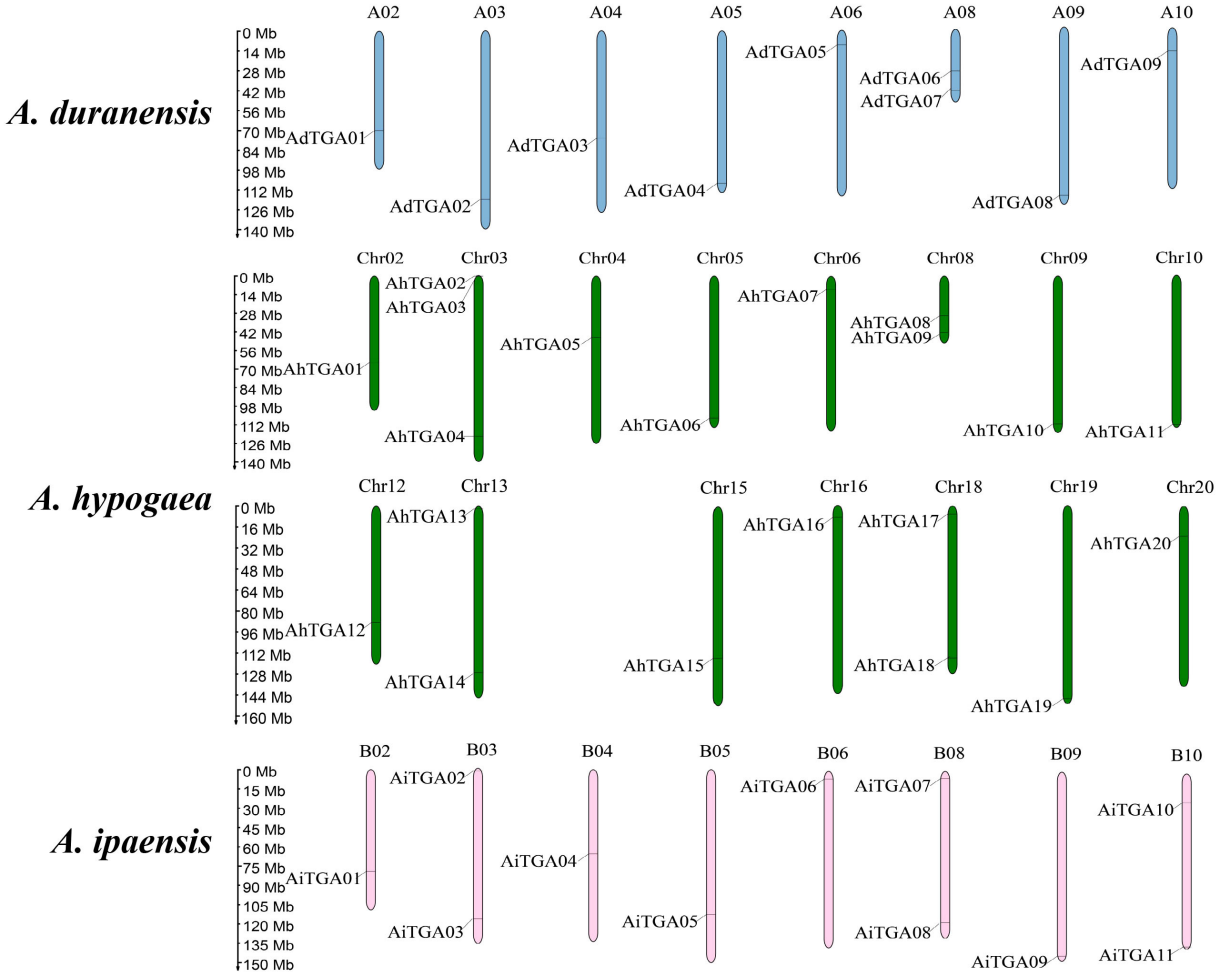
Distribution of *TGA* genes in three *Arachis* species genome. The chromosomes of *A. duranensis*, *A. ipaensis* and *A. hypogaea* were shown with pink, green and blue colors, respectively. Chromosome size is indicated by its relative length. The scale on the left is shown in megabases (Mb).

**Figure 4:**
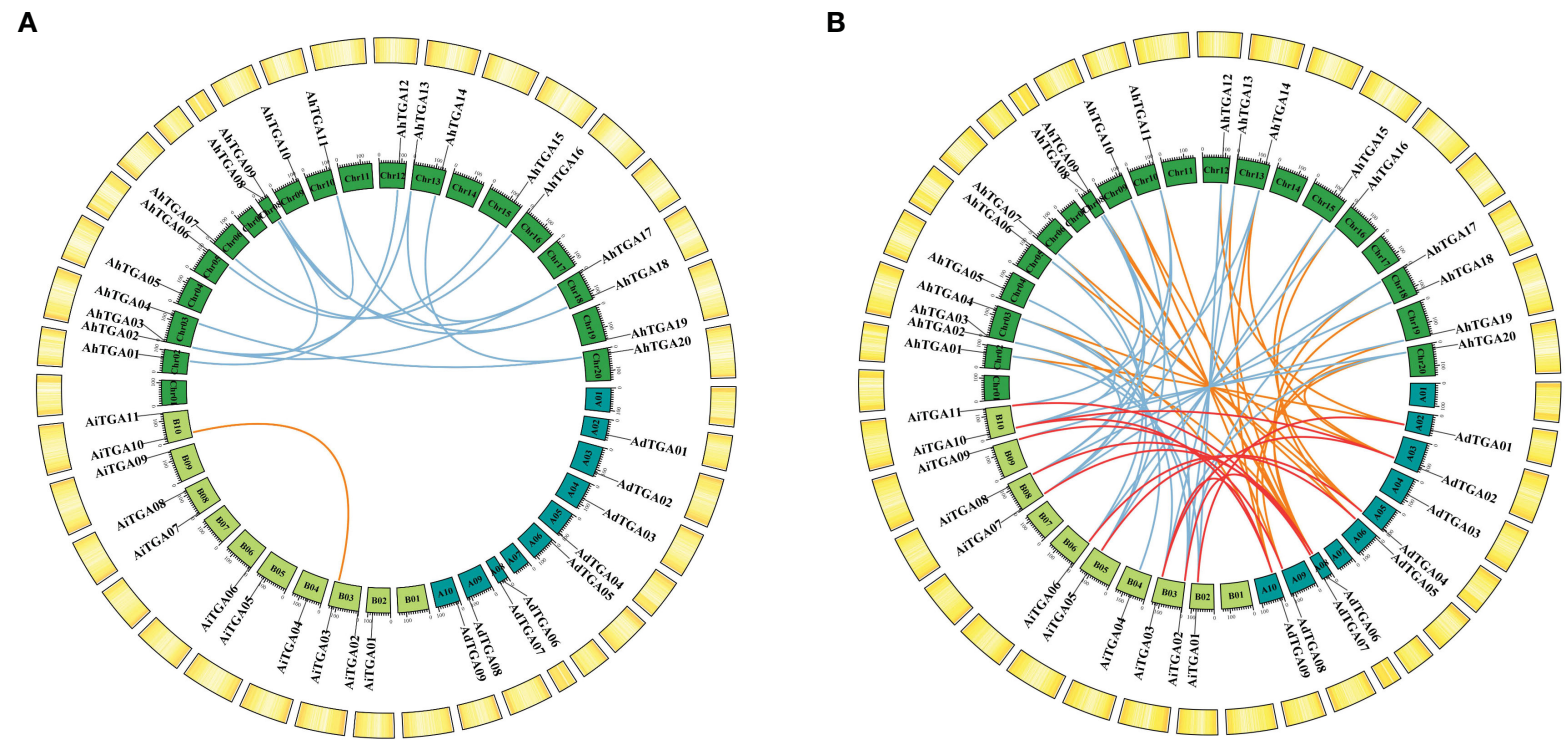
Syntenic analysis of *TGA* genes between cultivated peanuts and other plants. **(A)** Syntenic relationships of *TGA* genes with in cultivated peanut and two wild diploid peanuts, respectively. **(B)** Syntenic connections among *TGA* genes across *A*. *duranensis*, *A*. *ipaensis*, and *A. hypogaea* were examined. The chromosomes of *A*. *duranensis*, *A*. *ipaensis*, and *A*. *hypogaea* are depicted in dark green, light green, and green colors, respectively. Putative homologous *TGA* genes are indicated by lines of varying colors.

The *AhTGA* genes, comprising a total of 20 genes, are distributed across 15 chromosomes in the cultivated peanut genome. Similarly, the wild peanut species *A. duranensis* and *A. ipaensis* possess 20 *TGA* genes, which are located on eight chromosomes, respectively (**Figure 3**). While the chromosomal locations of most *TGAs* in cultivated peanut remain consistent with those in wild peanut species, some *TGA* genes have undergone changes in their genomic positions, likely attributed to segmental duplication events within the cultivated peanut genome (**Figure 4A**). These genetic rearrangements contribute to the diversification of the *TGA* gene family in cultivated peanut. To gain insights into the evolutionary mechanisms governing the *AhTGA* gene family, gene duplication events within the *Arachis* species were analyzed using MCScanx, leading to the identification of several duplication events (**Figure 4**; **Supplementary Table 3**). Notably, cultivated peanut lacks tandem duplications among its *TGA* genes, but it does exhibit 14 gene pairs for segment duplication. In contrast, only one pair of segmental duplication was detected in the wild peanut species *A. ipaensis*, while *A. duranensis* did not display any duplication events (**Figure 4A**). Furthermore, collinearity analysis revealed that 23 gene pairs were shared between *A. hypogaea* and *A. duranensis*, while 28 gene pairs were common to *A. hypogaea* and *A. ipaensis*. Additionally, 12 collinear gene pairs were identified between *A. duranensis* and *A. ipaensis* (**Figure 4B**). These findings emphasize a robust collinearity relationship between wild and cultivated peanut species.

### Synteny analysis of *TGA* genes among various species

**Figure 5:**
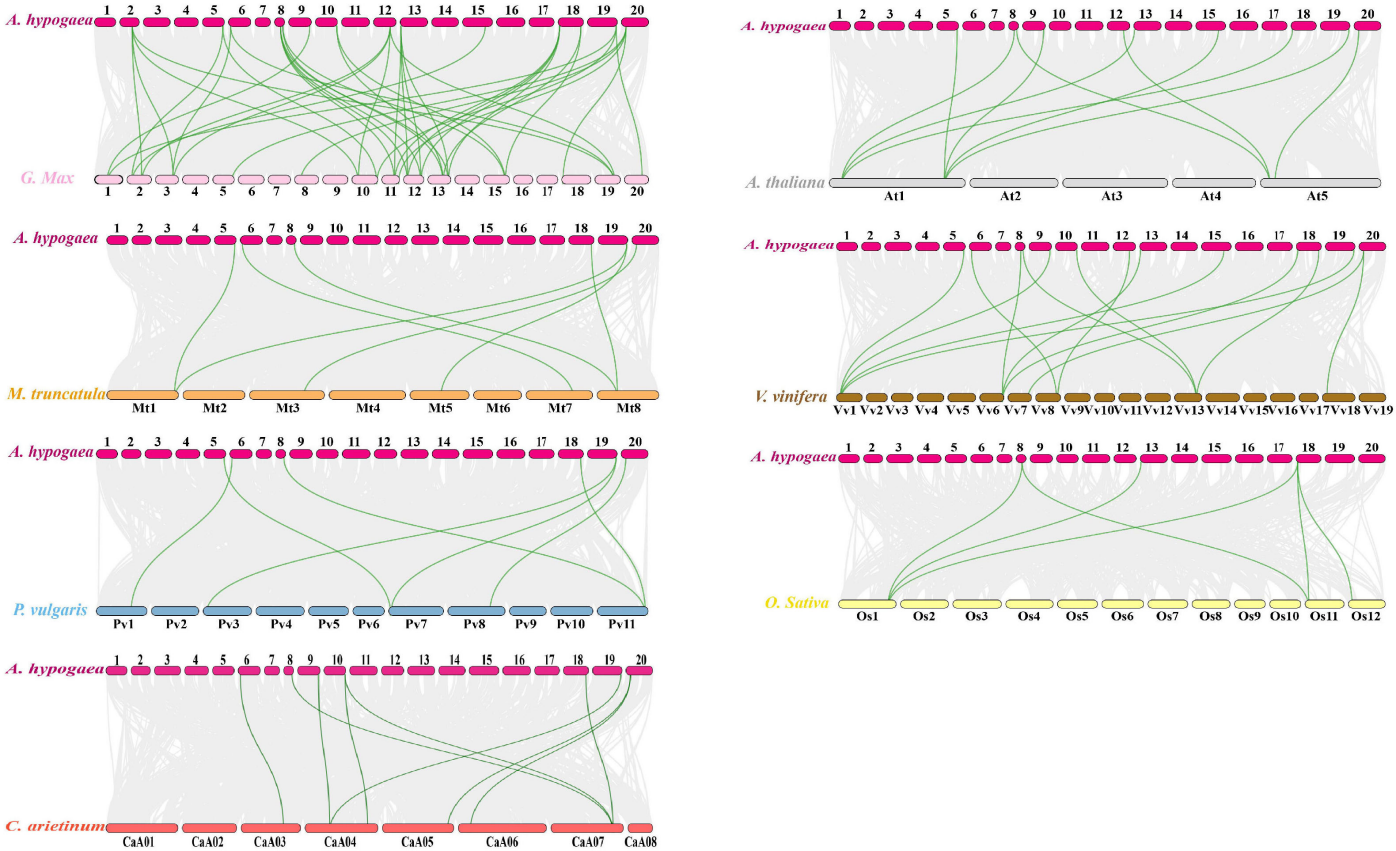
Syntenic analysis of *TGA* genes between cultivated peanuts and other plants, including soybean, chickpea, alfalfa, common beans, *Arabidopsis*, grapes, rice, sorghum and maize. Gray lines in the background indicated the collinear blocks with in *A. hypogaea* and other plant genomes, while the syntenic *TGA* gene pairs are linked with green lines.

Orthologous gene pairs were identified between *TGA* genes in cultivated peanut and those in other plants, including soybean, chickpea, alfalfa, common bean, *Arabidopsis*, grape, rice, sorghum, and maize (**Figure 5**; **Supplementary Table 3**). There were 39, 7, 9, and 7 gene pairs between cultivated peanut and other legumes, including soybean, alfalfa, chickpea and common bean, respectively; 14 and 10 gene pairs were found between cultivated peanut and the dicotyledonous plants grape and *Arabidopsis*, respectively; 6 gene pairs were identified between cultivated peanut and the monocotyledonous plant rice, while there were no orthologous gene pairs between cultivated peanut and the monocotyledonous plants sorghum and maize. Collinearity analysis revealed that the relationship of *TGA* genes between cultivated peanut and dicotyledonous plants was closer than that with monocotyledonous plants, with the closest relationship found with soybean.

Comparison of orthologous gene pairs revealed differences in the synteny relationships of *AhTGA* genes with various species. For example, *AhTGA20* in Group I showed the highest number of orthologous gene pairs (15 pairs) with other species, followed by *AhTGA17* in Group IV and *AhTGA19* in Group V, each having 12 pairs of orthologous gene pairs. *AhTGA08*, *AhTGA09*, and *AhTGA13* in had 10-11 pairs of orthologous gene pairs with other species. These results indicate that the synteny modules containing these genes are highly conserved in different species genomes. There were differences in the synteny of *AhTGAs*, as some *TGA* genes’ synteny modules were only present within the three peanut genomes, such as *AhTGA16*, *AhTGA05*, *AhTGA04*, *AhTGA14*, and *AhTGA02*. Other *AhTGAs* showed synteny relationships with other legume crops, while cultivated peanuts and rice, a monocot, showed synteny only in Group V. These results suggest that different *AhTGA* genes gradually evolved with the replication of synteny modules during the evolutionary process.

To analyze the evolutionary selection pressure on *AhTGA* genes, we calculated the Ka/Ks ratio of *TGA* gene pairs. Except for 14 collinear gene pairs that could not be calculated, the Ka/Ks ratios of the remaining 131 collinear gene pairs between cultivated peanut and other species were all less than 1, indicating that the *AhTGA* genes were mainly subject to purifying selection during the evolutionary process (**Supplementary Table 4**).

### Promoter analysis of *AhTGAs*

**Figure 6:**
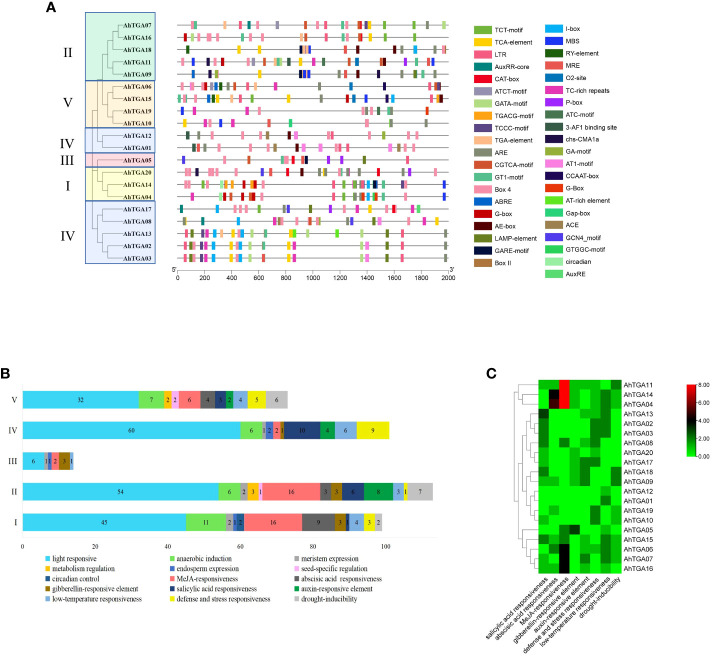
Analysis of the *cis-*acting elements in the promoter regions of 20 *AhTGA* genes. **(A)** Distribution of *cis-*elements in the promoters of *AhTGAs*. Gray lines indicate the promoters. *Cis-*elements differing in function are color-coded accordingly. **(B)** Functional statistics of *cis-*elements in the promoters within different groupings. **(C)** *Cis-*acting elements of *AhTGA* genes responded to cold, drought stress and hormone treatment.

Promoter analysis plays a crucial role in unraveling the transcriptional regulation and potential functions of peanut *TGA* genes. To gain insights into these aspects, we conducted an investigation by submitting the 2000 bp regulatory region upstream of the ATG (promoter) to the PlantCARE database, which allowed us to detect *cis-*acting elements. Remarkably, a total of 860 *cis-*acting elements, encompassing 43 different types with potential functions, were successfully predicted (**Figure 6A**; **Supplementary Table 5**). Among the predicted *cis-*acting elements, 29 types were associated with growth and development, 9 were related to hormones, and 5 were associated with stress responses. Notably, a significant portion of the growth and development-related elements were light-responsive components, with a total count of 223. Hormone-responsive elements accounted for 102 instances, including 42 JA-responsive elements, 20 SA-responsive elements, 16 ABA-responsive elements, 10 GA-responsive elements, and 14 auxin-responsive elements. Furthermore, the promoter regions of *AhTGA* genes exhibited a clustering pattern into five distinct clades, which was consistent with the protein sequence analysis. Intriguingly, variations were observed in the composition of *cis-*acting elements present among the different clusters (**Figure 6B**). A thorough analysis of *cis-*acting elements in the *AhTGA* genes responding to hormones and abiotic stresses such as low temperature and drought has been conducted (**Figure 6C**). The hormone responses are primarily focused on MeJA, SA, ABA, GA, and auxins. Variations in the distribution of *cis-*acting elements in different genes have been observed; for instance, MeJA-related elements are found to be more abundant in *AhTGA04*, *AhTGA11*, and *AhTGA14*, while SA-responsive elements are more prevalent in *AhTGA02*, *AhTGA03*, and *AhTGA13*. Concerning stress responses, it has been noted that most *AhTGA* genes contain 1-3 *cis-*acting elements related to low temperature or drought, but some genes, such as *AhTGA08*, *AhTGA17*, and *AhTGA20*, are not associated with these elements related to low temperature and drought.This finding suggests that different types of *TGA* genes interact with specific transcription factors, enabling their participation in diverse regulatory pathways.

### Protein-protein and miRNA-genes regulatory networks prediction for AhTGAs

**Figure 7:**
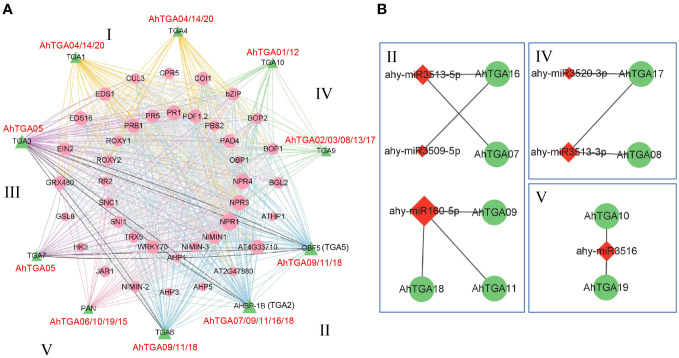
Interactions and regulatory associations involving peanut *TGA* genes with other proteins and miRNAs were explored. **(A)** A protein-protein interaction network involving peanut TGA proteins and other interacting proteins is presented. Peanut TGA proteins are represented by green triangles, while other proteins interacting with *AhTGAs* are depicted as pink circles. Interaction relationships are categorized into five groups based on evolutionary connections, denoted by different colored lines. **(B)** A regulatory network illustrates the potential miRNAs and their corresponding targeted AhTGAs. MiRNAs are represented by red lozenges, and the targeted AhTGAs are denoted by green circles. The presumed regulatory associations between miRNAs and their targeted AhTGAs are depicted as grey lines.

To gain further insights into the potential functions, signal transduction, and metabolic pathways of AhTGA members, we constructed a protein-protein interaction (PPI) network (**Figure 7A**). The analysis revealed that all 20 *AhTGA* genes exhibited orthologous relationships with 10 *Arabidopsis TGAs* and interacted with 40 functional proteins (**Figure 7A**; **Supplementary Table 6**). Notably, the proteins interacting with AhTGAs encompassed stress-responsive and pathogen defense-related proteins, such as NPR1, PR1, NIMIN1, and WRKY70. Additionally, several proteins involved in reproductive growth and flower organ development, including COI1, ROXY1, BOP1, and BOP2, were identified based on gene ontology (GO) information (**Supplementary Figure 3**).

Based on the functional classification of genes, all 20 *AhTGA* members, across the five groups, were found to be involved in the interaction with stress-responsive proteins. Furthermore, 16 AhTGA members in the four groups, except Group V, interacted with proteins associated with plant pathogen defense. Moreover, 9 members from Group I, II, and Group III were implicated in responding to environmental stress. These findings collectively indicate that the majority of *AhTGA* genes play a crucial role in disease resistance and stress response.

In addition to protein-protein interactions, we performed miRNA target prediction for *AhTGA* genes. The analysis revealed that 9 *AhTGA* genes were targeted by 6 miRNAs belonging to 6 different families (**Figure 7B**; **Supplementary Table 7**). Most miRNAs were found to target only one or two *AhTGA* genes, except for ahy-miR160-5p, which targeted 3 genes (*AhTGA11*, *AhTGA18*, and *AhTGA09*). Interestingly, specific groups of *AhTGA* genes were regulated by distinct miRNAs. For instance, in Group II, 5 *TGA* genes were targeted by 3 miRNAs, namely ahy-miR3513-5p, ahy-miR3509-5p, and ahy-miR160-5p. Additionally, *AhTGA17* and *AhTGA08* in Group IV were regulated by ahy-miR3509-5p and ahy-miR3513-3p, respectively. *AhTGA10* and *AhTGA19* in Group V were targeted by ahy-miR3516. Notably, no miRNA regulation was observed in Group III and Group I. These results suggest that miRNAs may play a crucial role in the molecular regulation of *AhTGA* genes. Moreover, the regulation of *AhTGA* genes by different types of miRNAs in different groups may contribute to variations in their expression levels, thereby influencing their functions.

### Expression patterns of *AhTGA* genes in various tissues and under different hormone and abiotic stress conditions

**Figure 8:**
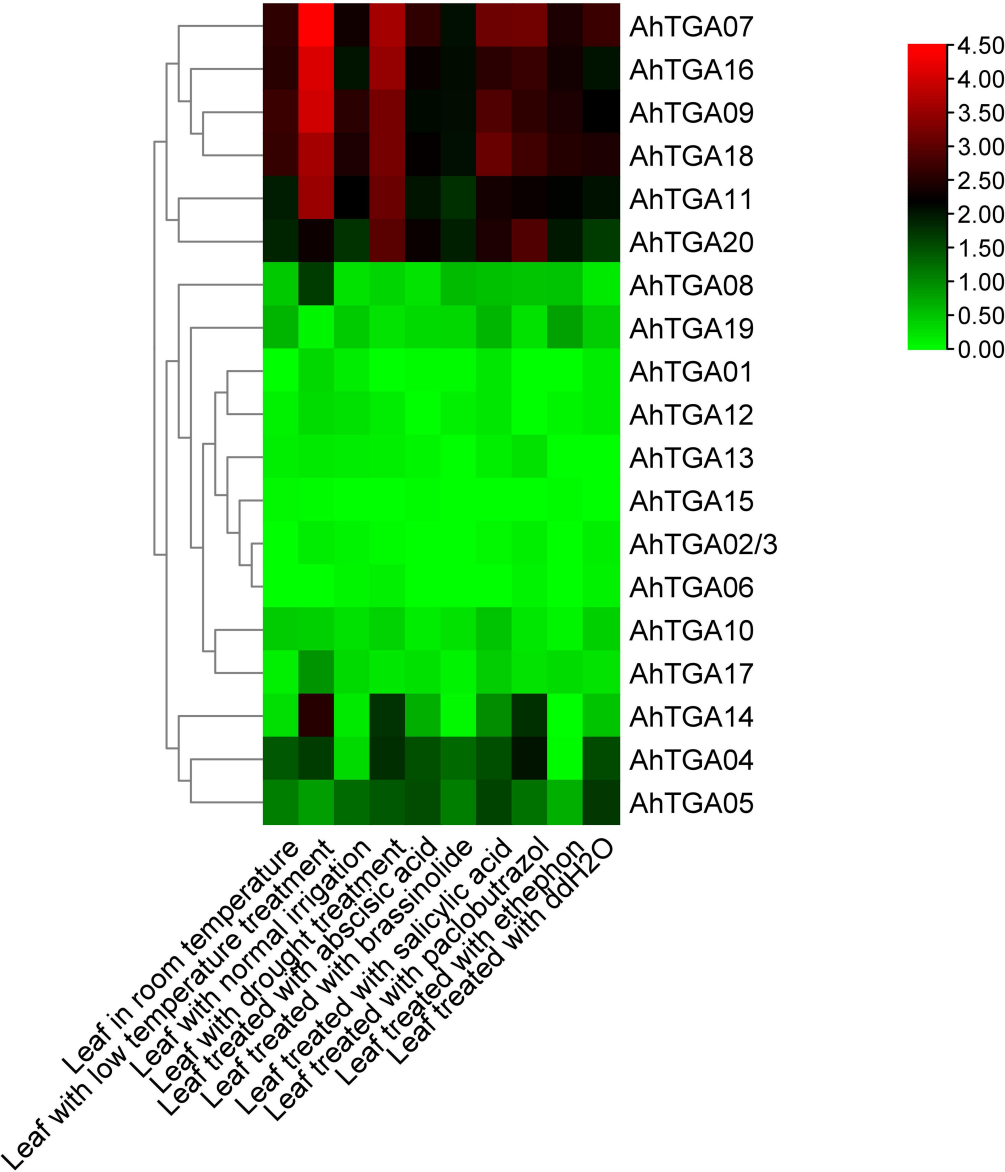
Expression profiles of 20 *AhTGAs* under different hormone and abiotic stress conditions. Heatmap clustering was based on the FPKM values of *AhTGA* genes following hormone and stress condition treatments. Log2-transformed values are used in a color-coded heatmap, with bars representing normalized FPKM (Log2) expression levels, and red for higher expression, green means low.

*AhTGA* gene expression patterns were investigated using RNA-Seq data, encompassing various developmental stages and tissues. Unique expression profiles for 20 *AhTGA* genes were revealed in the analysis of 22 tissues. Similar expression in orthologous pairs from A and B subgenomes was observed due to mRNA and promoter sequence similarity (**Supplementary Figure 4**). Expression was detected in all 22 tissues for *AhTGA07*, *AhTGA16*, *AhTGA11*, *AhTGA09*, *AhTGA18*, and *AhTGA20*, indicating their involvement throughout the peanut life cycle. Certain genes, such as *AhTGA01*, *AhTGA06*, *AhTGA08*, *AhTGA12*, *AhTGA13*, *AhTGA15*, and *AhTGA17*, showed higher expression in roots, nodules, and reproductive organs, suggesting roles in peanut reproductive development and underground growth. Similar expression levels among homologous genes indicated functional redundancy. Orthologous *AhTGA*s were identified in the reference genome Shitouqi (**Supplementary Table 8**) (Zhuang et al., 2019). The transcriptome-based expression patterns of orthologs under hormone, low-temperature, and drought treatments were determined. The results showed that, regardless of low-temperature or drought treatments, the expression levels of five *TGA* genes in subgroup II, *AhTGA07*, *AhTGA09*, *AhTGA11*, *AhTGA16*, and *AhTGA18*, significantly increased. In subgroup I and III, *AhTGA04*, *AhTGA05*, *AhTGA14*, and *AhTGA20* also exhibited significant upregulation under stress conditions but at lower expression levels than genes in subgroup II (**Figure 8**). In contrast, under different hormone treatments, genes in subgroups I, II, and III showed significantly higher expression compared to others. Combining tissue-specific expression with stress and hormone responses, the five *TGA* genes in subgroup II, *AhTGA07*, *AhTGA09*, *AhTGA11*, *AhTGA16*, and *AhTGA18*, displayed higher expression levels throughout the entire developmental stages of peanut and significantly increased expression under stress and hormone treatments such as salicylic acid, indicating their crucial roles in peanut growth, development, and stress regulation.

### Expression patterns of *AhTGAs* under abiotic stresses and diverse hormone treatment

**Figure 9:**
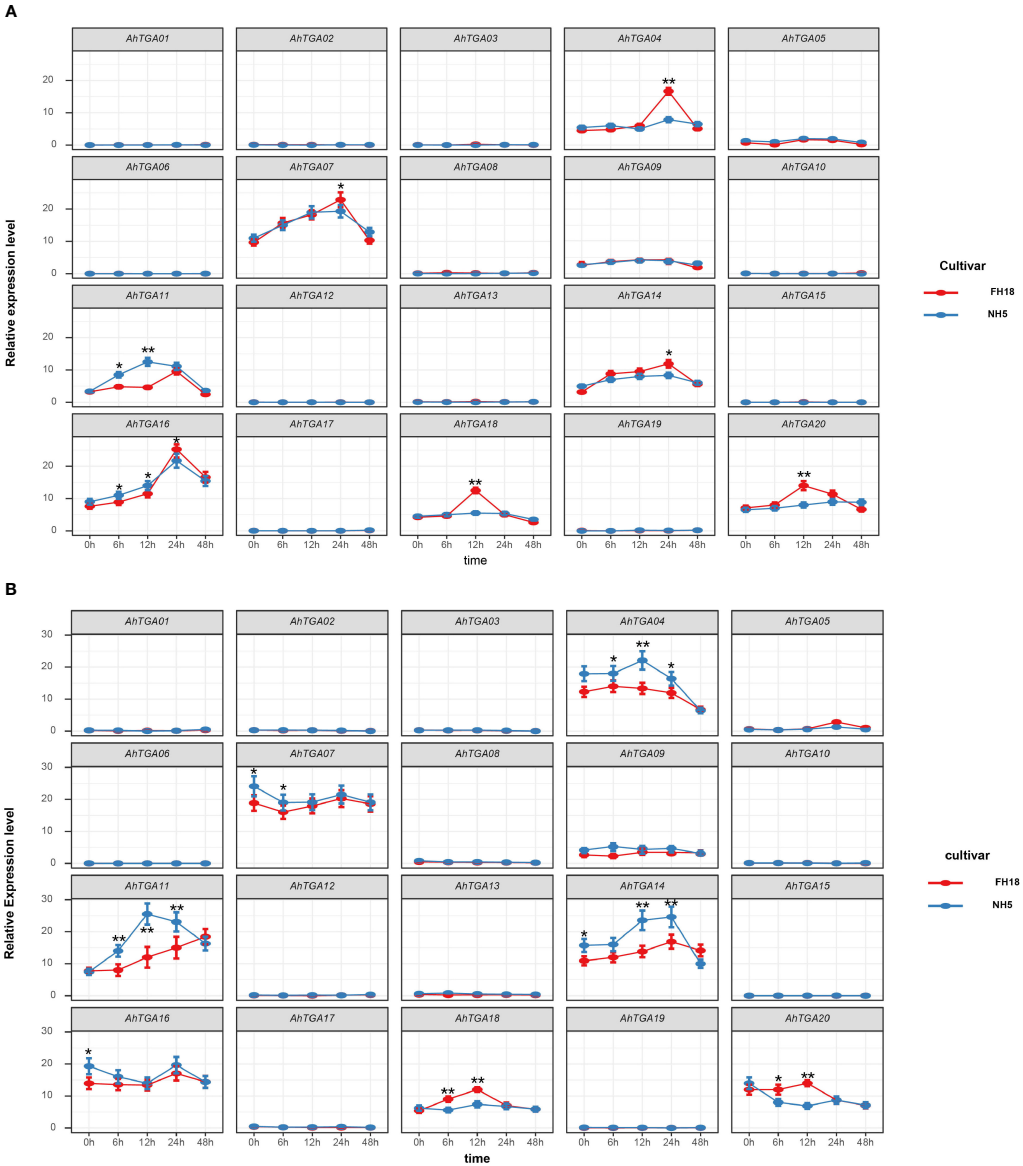
Expression patterns of the *AhTGA* genes in NH5 and FH18 under drought and low-temperature treatments. **(A)** Relative expression levels of the *AhTGA* gene in the cold-sensitive variety FH18 and the cold-tolerant variety NH5. **(B)** Relative expression levels of the *AhTGA* genes in the drought-sensitive variety FH18 and the drought-tolerant variety NH5. Student’s *t*-test was used to detect differences between the two varieties at the same time point (*, *P* < 0.05; **, *P* < 0.01).

To investigate the response of *AhTGA* genes to abiotic stresses and hormone treatments, we performed qPCR analysis on two peanut varieties, NH5 (tolerant) and FH18 (sensitive), under low temperature and drought stress conditions (**Figure 9**). The expression levels of selected *TGA* genes were measured at five different time points. The primers used for qPCR are listed in **Supplementary Table 9**. Our results revealed that several genes, including *AhTGA01*, *AhTGA02*, *AhTGA03*, *AhTGA06*, *AhTGA08*, *AhTGA09*, *AhTGA10*, *AhTGA12*, *AhTGA13*, *AhTGA15*, *AhTGA17*, and *AhTGA19*, exhibited relatively low expression levels in both NH5 and FH18 under low temperature and drought stress treatments. Furthermore, no significant changes in expression levels were observed across the five time points, suggesting that these genes may not be involved in stress responses or may lack functional roles under these conditions.

Under low temperature conditions, the expression levels of *AhTGA04*, *AhTGA07*, *AhTGA11*, *AhTGA14*, *AhTGA16*, *AhTGA18*, and *AhTGA20* exhibited significant changes over time. Some genes showed differential expression patterns between cold-tolerant and cold-sensitive varieties. For instance, *AhTGA04* and *AhTGA14* exhibited significantly higher expression levels in FH18 than in NH5 at 24 hours, while *AhTGA18* and *AhTGA20* displayed significantly higher expression levels in FH18 than in NH5 at 12 hours. These findings suggest that these genes have distinct expression patterns between cold-tolerant and cold-sensitive varieties and may play roles in the molecular regulation of peanut cold tolerance (**Figure 9A**). Similar expression patterns were observed under drought stress conditions. *AhTGA04*, *AhTGA07*, *AhTGA11*, *AhTGA14*, *AhTGA16*, *AhTGA18*, and *AhTGA20* also displayed higher expression levels, but with differences in expression patterns between FH18 and NH5. For instance, *AhTGA04* and *AhTGA14* exhibited opposite expression patterns compared to those under low temperature conditions, showing significantly upregulated expression in FH18. On the other hand, *AhTGA18* and *AhTGA20* displayed similar expression patterns to those under low temperature conditions, with significantly higher expression levels in FH18 than in NH5 at 12 hours (**Figure 9B**).

**Figure 10:**
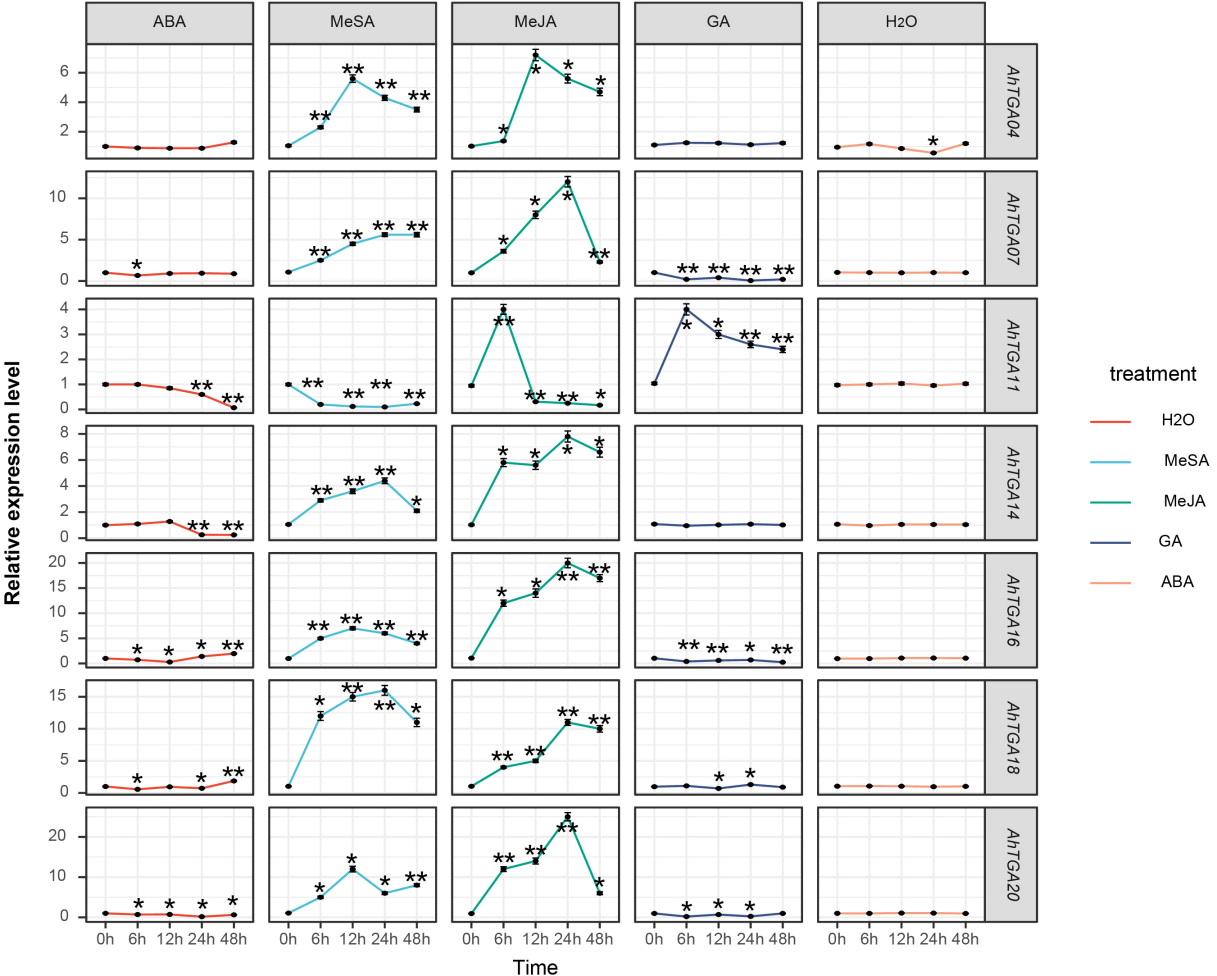
The expression levels of the *AhTGA* genes under different hormone treatments were examined in. The differential expression levels of *AhTGA* in FH18 after hormone treatment were analyzed using ANOVA and Dunnett’ s multiple comparison method, with the 0h time point used as the control group. Significant differences were indicated by ** (*P* < 0.01).

To elucidate the involvement of different hormone regulatory mechanisms in *AhTGA* genes, we treated the low temperature and drought-sensitive variety FH18 with exogenous hormones and analyzed the expression characteristics of selected *TGA* genes (**Figure 10**). Seven Genes that displayed significant expression level differences under low temperature and drought conditions were selected for analysis under hormone treatments. Within 48 hours of distilled water spray treatment, the expression levels of seven genes did not show significant changes. However, after hormone treatments, the expression levels of *AhTGA* genes exhibited significant alterations, particularly in response to MeJA and MeSA treatments compared to ABA and GA treatments. With the exception of *AhTGA*, the expression levels of other *AhTGA* genes showed an increase followed by a decrease. Notably, the expression level of *AhTGA* treated with salicylic acid decreased significantly, whereas the expression levels of *AhTGA* genes treated with other hormones initially increased and then decreased. Following exogenous ABA treatment, the expression levels of *AhTGA11* and *AhTGA14* decreased, while exogenous GA treatment led to decreased expression levels of *AhTGA* genes, with AhTGA11 displaying an initial increase followed by a decrease. These findings suggest that *AhTGA* genes are involved in the regulation of different hormone pathways, with the salicylic acid and jasmonic acid pathways showing the most significant regulation (**Figure 10**).

### Overexpression of *AhTGA11* confers cold and drought stress in transgenic *Arabidopsis*

**Figure 11:**
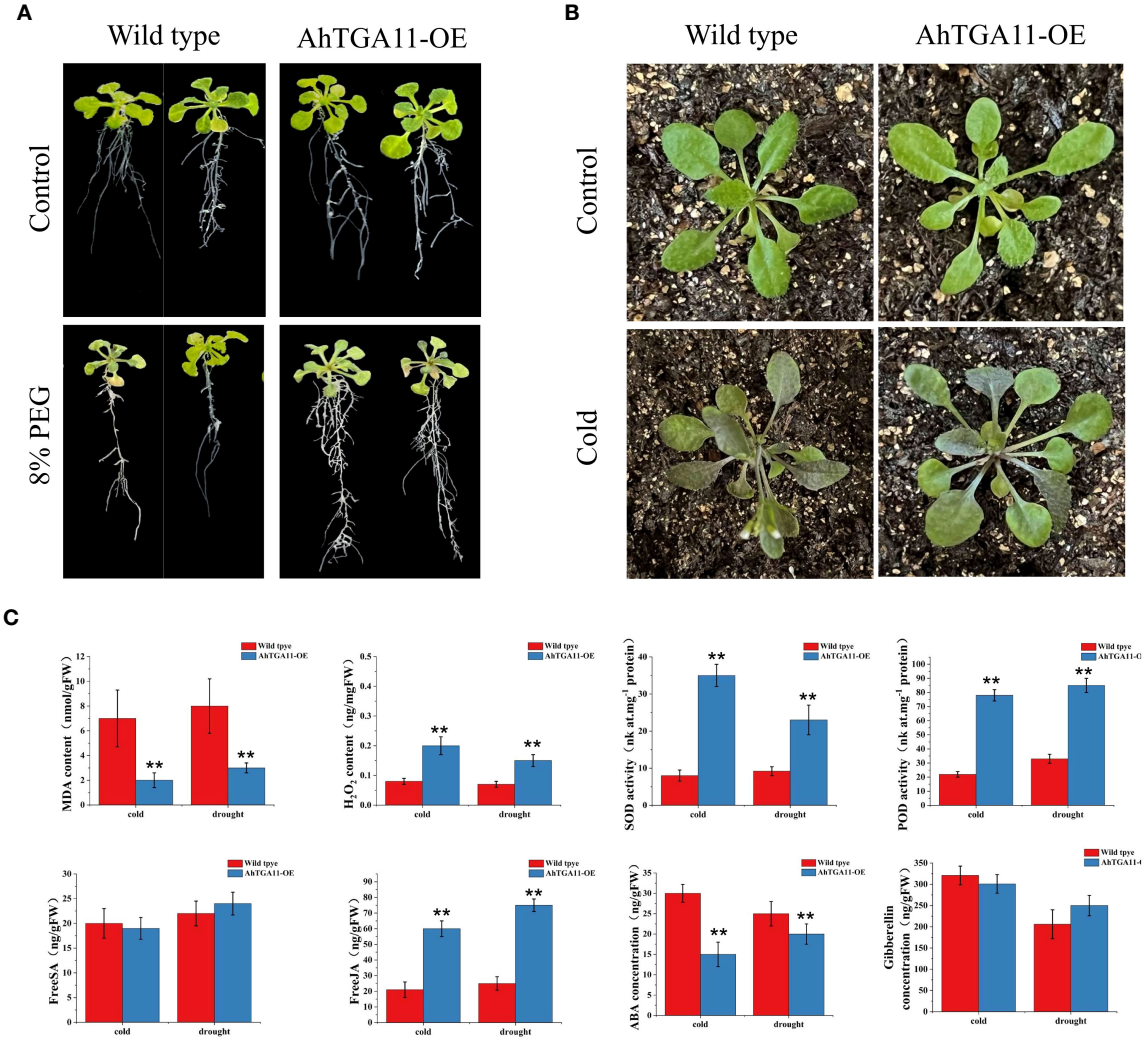
The phenotypic and physiological changes in AhTGA11-overexpressing *Arabidopsis* plants (AhTGA11-OE) and their wild-type under low temperature and drought stress conditions. **(A)** Phenotypic changes in AhTGA11-OE plants and their wild-type controls following 5 days of treatment with 8% PEG. **(B)** Eighteen-day-old seedlings of AhTGA11-OE plants and their wild types were subjected to 5 days of treatment at 4°C. **(C)** Physiological parameters and endogenous hormone alterations in AhTGA11-OE and wild-type plants under low-temperature and drought treatments. The data is expressed as the mean ± standard deviation (n = 3), and asterisks denote significant distinctions between transgenic plants and wild-type plants subjected to the same treatment, as determined by *t*-tests (** *P* < 0.01).

Due to its significant responses under both abiotic stress and hormonal treatments, as well as differential expression patterns observed between cold and drought-tolerant genotypes versus sensitive genotypes, *AhTGA11* is hypothesized to be a key factor in abiotic stress and hormone regulatory pathways. Therefore, *AhTGA11* was transformed into *Arabidopsis* and its gene function was identified in transgenic plants of the T_3_ generation. After amplification with the primers AhTGA11-F/R, the CDS sequence of *AhTGA11* was sequenced and found to be consistent with the reference genome sequence (**Supplementary Table 10**; **Supplementary Figure 5**). Following insertion into a vector under the control of the 35S promoter, *Arabidopsis* was transformed. Under non-PEG treatment conditions, the wild-type and AhTGA11-OE phenotypes were similar, whereas under 8% PEG treatment, the wild-type *Arabidopsis* plants exhibited wilting, with significantly reduced root length compared to the overexpressing AhTGA11-OE plants (**Figure 11A**). Under low-temperature conditions, the leaves of wild-type plants appeared water-soaked (**Figure 11B**). Physiological and endogenous hormone measurements of wild-type and *AhTGA11*-overexpressing plants under low-temperature and drought treatment conditions revealed that in overexpressing plants, root tissue H_2_O_2_ content, POD activity, and SOD activity were significantly higher than in wild-type plants, while MDA content was significantly lower than in wild-type (**Figure 11C**). Hormone measurements for four stress-related endogenous hormones showed that in overexpressing plants, JA content was significantly higher than in the wild-type, whereas ABA content was significantly lower than in the wild-type, with no significant differences in SA and GA. These results indicate that *AhTGA11* likely plays a positive regulatory role in drought and low-temperature stress in plants and is involved in mediating ABA and JA-related pathways.

### *AhTGA11* enhances drought resistance in transgenic soybean hairy roots

**Figure 12:**
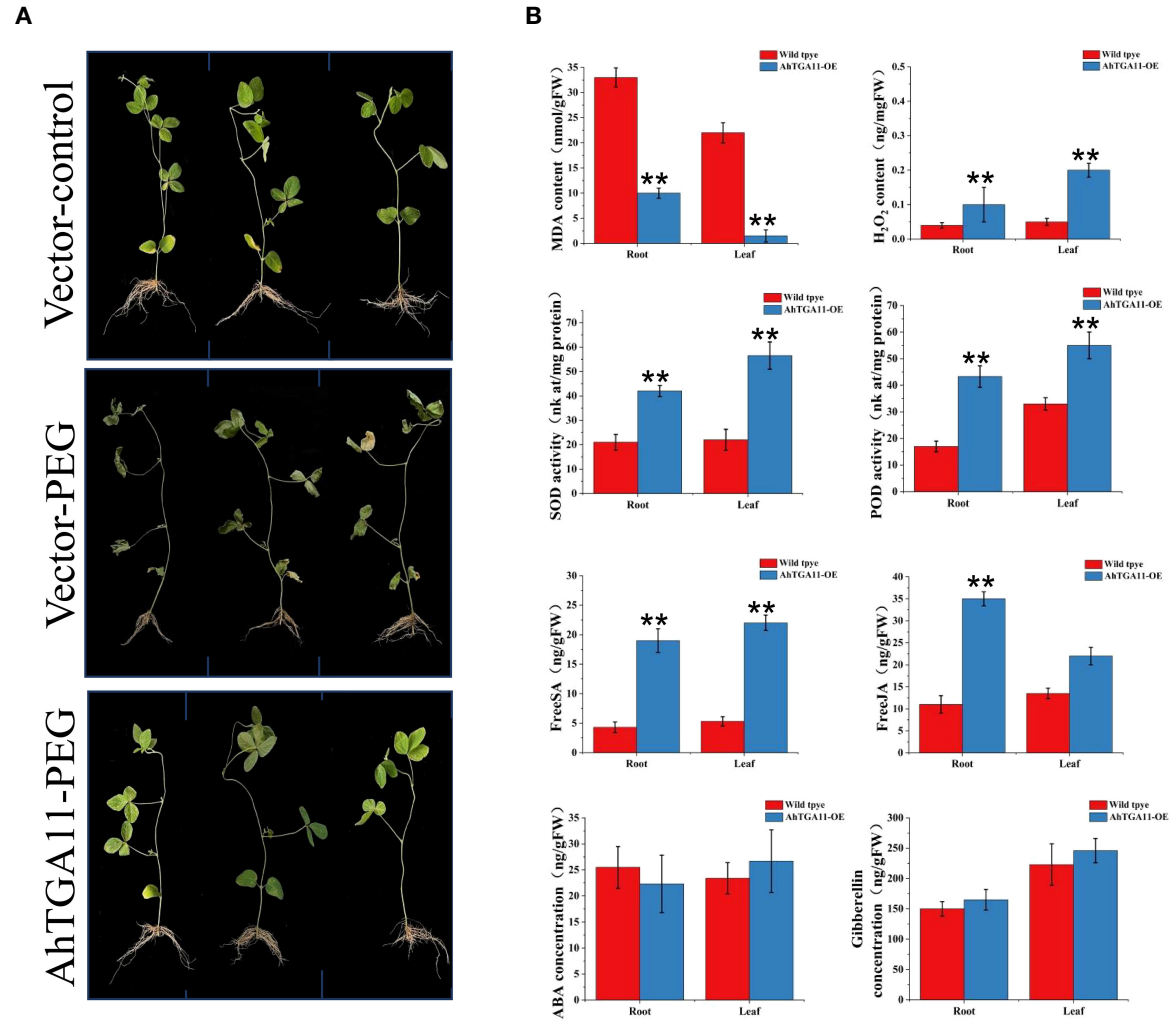
Phenotypes of *AhTGA11* transgenic soybean hairy roots under drought stress. **(A)** Phenotypic differences between *AhTGA11* transgenic soybean hair roots, empty vector transgenic soybean hair roots under PEG treatment, and their empty vector controls. **(B)** Physiological and endogenous hormone changes in *AhTGA11* transgenic soybean hair roots and leaves without transgenic components under drought treatment. The data is expressed as the mean ± standard deviation (n = 3), and asterisks denote significant distinctions between transgenic plants and wild-type plants subjected to the same treatment, as determined by *t*-tests (* *P* < 0.05; ** *P* < 0.01).

*AhTGA11*’s sensitivity to drought stress was assessed with a focus on the root region, and this assessment was conducted through *Agrobacterium rhizogenes*-mediated transformation of soybean hairy roots. After 4 days of exposure to 8% PEG treatment, it was observed that overexpressing *AhTGA11* did not exhibit significant changes when compared to transgenic hairy soybean roots containing an empty vector. In contrast, transgenic hairy roots containing an empty vector displayed symptoms of drought stress under PEG treatment (**Figure 12A**). Root tissue of overexpressing plants showed significantly higher levels of H_2_O_2_, POD activity, and SOD activity compared to control plants, while MDA content was significantly lower in overexpressing plants (**Figure 12B**). These trends were consistent between root tissue and non-transgenic leaf tissue. Furthermore, both root and leaf tissues of overexpressing plants exhibited significantly higher levels of endogenous SA and JA compared to control plants. However, there was no significant difference in GA content between overexpressing and wild-type plants in both root and leaf tissues.

## Discussion

Investigating gene families of different transcription factors is of great significance for uncovering the functions of key plant genes and understanding the intricate regulatory networks governing plant processes (Feng et al., 2020; Dong et al., 2023). In recent years, extensive studies have focused on elucidating the functions of transcription factor families in peanut (*Arachis hypogaea*), such as the *AP2/ERF*, *WRKY* and *NAC* families (Song et al., 2016; Yuan et al., 2020; Cui et al., 2021). These studies have shed light on the roles of these transcription factors in peanut growth, development, and stress responses. However, despite the importance of the *TGA* transcription factor in other plant species, such as *Arabidopsis* (Kim et al., 2022), rice (Ueda et al., 2020) and soybean (Li et al., 2019a), little is known about the *TGA* transcription factors in peanut. To address this knowledge gap, the present study aimed to identify and characterize the *TGA* transcription factors in peanut and explore their potential roles in peanut growth, development, and stress responses.

A total of 20 *TGA* genes were identified in cultivated peanut, while 9 and 11 *TGA* genes were identified in wild diploid peanuts, respectively. The majority of wild peanuts exhibit a correspondence between the number of *TGA* genes, their chromosomal locations, and physicochemical properties with the *TGA* genes in the subgenomes A and B of cultivated peanut. This alignment significantly improves the accuracy of *AhTGA* gene identification. The identified *AhTGA* genes all contained representative domains, bZIP and DOG1 (**Figure 2D**). *TGA* genes belong to the D branch of the *bZIP* gene family, and the number of genes identified in the D branch of *bZIP* in wild diploid peanuts differed from the findings of this study (Wang et al., 2019c). In contrast, our study employed the latest version of the tetraploid cultivated peanut and diploid wild peanut genomes (Genome Assembly 2) data, allowing for a more efficient identification of peanut gene family members. This approach likely led to more comprehensive genome annotations (Bertioli et al., 2019) (https://www.peanutbase.org/download/). When comparing the identification results of the D group of *bZIP* genes in wild diploid peanuts from previous studies, we identified two additional *AdTGA* genes, *AdTGA02* and *AdTGA03*, and four *AiTGA* genes, *AiTGA03*, *AiTGA04*, *AiTGA08*, and *AiTGA09*. These genes were also retained in the cultivated peanut genome as *AhTGA04*, *AhTGA05*, *AhTGA14*, *AhTGA18*, and *AhTGA19*. Notably, *AhTGA04* and *AhTGA18* exhibited changes in expression levels under stress and hormone treatments in this study (**Figure 9**), while other genes also showed expression level variations in different tissues (**Supplementary Figure 4**). This suggests that these genes may have corresponding functions in the growth, development, and stress response of cultivated peanuts. Thus, the utilization of the updated cultivated peanut genome data in this study facilitated the exploration of *AhTGA* genes. The targeted exploration of *TGA* genes in the *bZIP* gene family using newly released genome data has also been reported in other plants such as soybean and kiwifruit (Li et al., 2019a; Jin et al., 2021; Liu et al., 2022b; Yue et al., 2023).

Understanding the gene structure is of significant importance in elucidating gene function (Wang et al., 2019a). The structural features of a gene, such as the arrangement of exons and introns, promoter regions, and conserved domains, provide valuable insights into its regulatory mechanisms and functional roles (Weake and Workman, 2010). The non-coding regions of *AhTGA* genes exhibit significant variation, particularly in the UTR regions and introns, while the coding structure remains relatively conserved (**Figure 2C**). The five most conserved motifs, Motif 1-5, are present in every *AhTGA* gene, comprising the key structural domains of bZIP and DOG1 (**Figures 2B, D**). Specific motifs unique to different groups are mainly located at the N-terminus, indicating a higher diversity in the N-terminal region of *TGA* genes, while the C-terminal region is more conserved (**Figure 2**). bZIP proteins are characterized by their conserved bZIP domain, responsible for DNA binding and dimerization. The bZIP domain shows high sequence identity among *Arabidopsis TGAs* and is conserved across plant species (Jindrich and Degnan, 2016). The DOG1 domain, found in the C-terminus of *TGAs*, plays a role in seed dormancy control and potentially modulates *TGA* activity (Magnani et al., 2014). The presence of conserved residues implicated in Calmodulin (CaM) binding suggests a potential interaction between DOG1 and CaM, linking *TGA* transcriptional regulation to calcium signaling (Sall et al., 2019). The N-terminus of *TGAs* exhibits high variability in sequence and length, likely contributing to their functional specificity in transcriptional regulation (Katagiri et al., 1989; Tomaz et al., 2022). The *TGA* genes in peanut also exhibit these structural characteristics, indicating the conservation of *TGA* gene structure and function across different species.

Gene duplication is a key driver of gene family formation (Flagel and Wendel, 2009). No tandem duplications were found in both wild diploid and cultivated peanuts. In the genome of wild diploid peanuts, only one segment duplication was identified. However, within *A. hypogaea* genome, 14 pairs of segment duplications were detected. The occurrence of segment duplications is primarily attributed to the homologous genes derived from the *A. duranensis* and *A. ipaensis*, resulting in transpositions between the A and B subgenomes of cultivated peanut. Consequently, this leads to locational differences, gene duplications, or deletions among orthologous genes from A and B subgenomes within cultivated peanuts (**Figure 3**). Similar results have been documented in other polyploid plant species, including hexaploid wheat (*Triticum aestivum*) and cotton (*Gossypium hirsutum*) (Yousafzai et al., 2010; Paterson et al., 2012). Homologous exchange, also known as recombination, plays a vital role in creating genetic diversity and facilitating the evolution of species. Therefore, the origin of *TGA* genes in cultivated peanuts is primarily due to whole-genome duplication during the formation of tetraploid cultivated peanuts, as well as transposition events between the A and B subgenomes. *TGA* genes in peanuts lack tandem duplication and primarily originate from the retention of orthologous genes during the species evolution. The results of the synteny analysis between *AhTGA* genes and other species showed that *AhTGA* genes within the same group share the same collinearity modules (**Figure 5**; **Supplementary Table 3**). There are significant differences in collinearity of *AhTGA* genes across different species. For instance, *AhTGA20* exhibits collinearity relationships in all dicotyledonous plants studied in this research, while *AhTGA02*, *AhTGA04*, *AhTGA05*, *AhTGA14*, and *AhTGA16* show collinearity only in *Arachis* species. This suggests that different *AhTGA* genes have diverged in their formation time during evolution. In addition, all *AhTGA* genes have undergone purifying selection, as indicated by the Ka/Ks ratio calculation resulting in values less than 1. Combined phylogenetic analysis, gene structure, and collinearity analysis reveal that *AhTGA* genes have similar structures within the same clade, showing close phylogenetic and collinearity relationships with other species. Therefore, the sequence and structural analysis of *AhTGA* genes suggest that they may have similar functions within the same clade while exhibiting significant differences between clusters.

The expression patterns of the *AhTGA* genes under hormonal and abiotic stress in peanuts were studied using transcriptome data and qPCR. Significant differences in the expression of three *AhTGA* genes (*AhTGA04*, *AhTGA14*, and *AhTGA20*) in Group I and four *AhTGA* genes (*AhTGA7*, *AhTGA11*, *AhTGA16*, and *AhTGA18*) in Group II were observed across different peanut tissues, hormonal treatments, and stress responses (**Figures 8**–**10**). *AhTGA04*, *AhTGA14*, and *AhTGA20*, belonging to the same branch as the *Arabidopsis TGA* genes *AtTGA01* and *AtTGA04*, are involved in SA signaling and contribute to plant resistance against pathogens through their interaction with *NPR1* (Gatz, 2013; Shearer et al., 2021). In terms of abiotic stress, overexpression of *AtTGA4* in *Arabidopsis* has been shown to enhance drought tolerance by improving nitrate transport and absorption (Zhong et al., 2015). Genetic transformation of soybean with the overexpressing group I *GmTGA15* gene has been found to increase drought resistance (Chen et al., 2021). *AhTGA04* and *AhTGA14* are homologous genes from the A and B subgenomes, and under low-temperature conditions, the cold-tolerant genotype NH5 exhibits lower expression levels compared to the sensitive genotype FH18, whereas under drought conditions, the expression pattern is reversed (**Figures 8**–**10**). In Clade II, *AhTGA07*, *AhTGA11*, *AhTGA16*, and *AhTGA18* exhibit higher expression levels than other *AhTGA* genes in peanut, and their expression patterns are altered under low temperature, drought, and hormone treatments. *AtTGA2*, *AtTGA5*, and *AtTGA6*, as Clade II genes, are essential for systemic acquired resistance in *Arabidopsis*, as they interact with *NPR1* and regulate the expression of pathogenesis-related (*PR*) genes and pathogen resistance (Zhang et al., 2003). Low temperature and drought stress can disrupt membrane lipid metabolism (Gigon et al., 2004; Zhang X. et al., 2019). *AtTGA2*, *AtTGA5*, and *AtTGA6* play critical roles in lipid stress responses (Mueller et al., 2008). These transcription factors are involved in both the SA-dependent SAR pathway against biotrophic pathogens and the JA-ethylene-dependent defense mechanism against necrotrophic pathogens (Zander et al., 2010; Zander et al., 2014). Interestingly, in this study, besides being regulated by JA and SA, we also found significant changes in the expression levels of *TGA* genes in response to exogenous ABA and GA treatments. While the ability of *TGA* genes to respond to ABA induction has been reported in soybean, the involvement of *TGA* genes in GA-related gene regulation is less explored (Li et al., 2019a). Therefore, further investigations are needed to elucidate the regulatory mechanisms of *AhTGA11* in response to GA induction (**Figure 10**).

*AhTGA11* was selected for functional validation in transgenic *Arabidopsis* and soybean hairy roots. The results demonstrated that *AhTGA11* effectively alleviates plant antioxidant activity under low-temperature and drought conditions, thereby mitigating abiotic stress. However, in terms of hormone regulation, there were differences in the overexpression of *AhTGA11* between transgenic *Arabidopsis* and soybean hairy roots. For instance, salicylic acid did not accumulate significantly in *Arabidopsis*, whereas ABA levels were significantly identified. Additionally, gibberellin levels did not accumulate in either transgenic soybean or *Arabidopsis*. This discrepancy may be attributed to the different genetic backgrounds of the two species, highlighting the complexity of *TGA* transcription factor regulation and the significant interspecies differences involved. The functional roles of *TGAs* from different clades have traditionally been associated with plant immunity for clades I, II, and III, while clades IV and V were initially implicated in developmental processes (Gatz, 2013). However, emerging evidence suggests that the functional division between clades is not as clear-cut, as studies have revealed their involvement in various biological processes. For instance, clade I *TGAs* have been shown to play a role in growth and development regulation (Li et al., 2019b; Wang et al., 2019b), and clade IV *TGAs* have been implicated in biotic stress responses (Venturuzzi et al., 2021). Functional analyses of *TGAs* across different clades have demonstrated their significance not only in biotic stress responses (Kesarwani et al., 2007; Sun et al., 2018) but also in the regulation of gene expression related to abiotic stress responses (Fang et al., 2017; Herrera-Vasquez et al., 2021), developmental processes (Maier et al., 2011), circadian rhythm (Zhou et al., 2015), detoxification (Fode et al., 2008; Mueller et al., 2008; Herrera-Vasquez et al., 2021), nitrate signaling (Alvarez et al., 2014; Canales et al., 2017), flowering (Thurow et al., 2005; Xu et al., 2021), and autophagy (Wang et al., 2020). Although we conducted in silico and expression patterns analysis of *AhTGA* genes, and functional identification of *AhTGA11* in other plants species, it is important to note that *TGA* genes hold a pivotal position in various facets of plant regulatory pathways, encompassing growth, development, and stress responses. As peanuts are a tetraploid crop with a complex genetic backgroud, this complexity may result in increased functional redundancy and evolutionary adaptations. Consequently, additional research efforts are warranted to thoroughly characterize the functions of each *AhTGA* gene in cultivated peanut.

## Conclusion

In our study, a comprehensive *in silico* analysis was conducted to investigate the *AhTGA* gene family in cultivated peanut, including evolutionary analysis, gene structure examination, identification of regulatory elements, prediction of protein-protein interactions, and identification of miRNA targets. A total of 20 *AhTGA* genes were identified and classified into five groups. Differential expression patterns of *AhTGA* genes in different tissues, under abiotic stress conditions such as low temperature and drought, and in response to hormonal stimuli were analyzed. *AhTGA11* was chosen for functional validation in transgenic *Arabidopsis* plants and soybean transgenic hairy roots. It was found to have a positive role on both low-temperature and drought responses, involving regulation by SA, JA, and ABA, mitigating the oxidative stress generated in plants due to stress. These findings provide valuable insights into the functional characterization of *AhTGA* genes and can guide the breeding of novel abiotic-resistant peanut varieties.

## Data availability statement

The original contributions presented in the study are included in the article/**Supplementary Material**. Further inquiries can be directed to the corresponding authors.

## Author contributions

CZ: Conceptualization, Data curation, Funding acquisition, Software, Validation, Writing – original draft, Writing – review & editing. YL: Data curation, Methodology, Software, Validation, Visualization, Writing – original draft, Writing – review & editing. ZL: Data curation, Software, Visualization, Writing – review & editing. XW: Methodology, Supervision, Writing – review & editing. CJ: Investigation, Methodology, Supervision, Writing – review & editing. XZ: Data curation, Investigation, Supervision, Writing – review & editing. SK: Formal Analysis, Investigation, Project administration, Writing – review & editing. XL: Formal Analysis, Investigation, Project administration, Supervision, Writing – review & editing. SZ: Project administration, Supervision, Writing – review & editing. JW: Data curation, Formal Analysis, Methodology, Writing – review & editing. HZ: Data curation, Software, Writing – review & editing. YH: Formal Analysis, Investigation, Supervision, Writing – review & editing. HY: Conceptualization, Funding acquisition, Resources, Supervision, Writing – review & editing. RX: Conceptualization, Funding acquisition, Resources, Supervision, Writing – review & editing.
