# Supplementary material for: Genome-wide analysis reveals regulatory mechanisms and expression patterns of TGA genes in peanut under abiotic stress and hormone treatments
Source: Front Plant Sci. 2023 Nov 21;14:1269200. doi: 10.3389/fpls.2023.1269200 (PMC10702600; doi:10.3389/fpls.2023.1269200)
Supplement: Supplementary file 1 [file DataSheet_1.zip › Data Sheet 1 (8)/Figure S1.pdf]

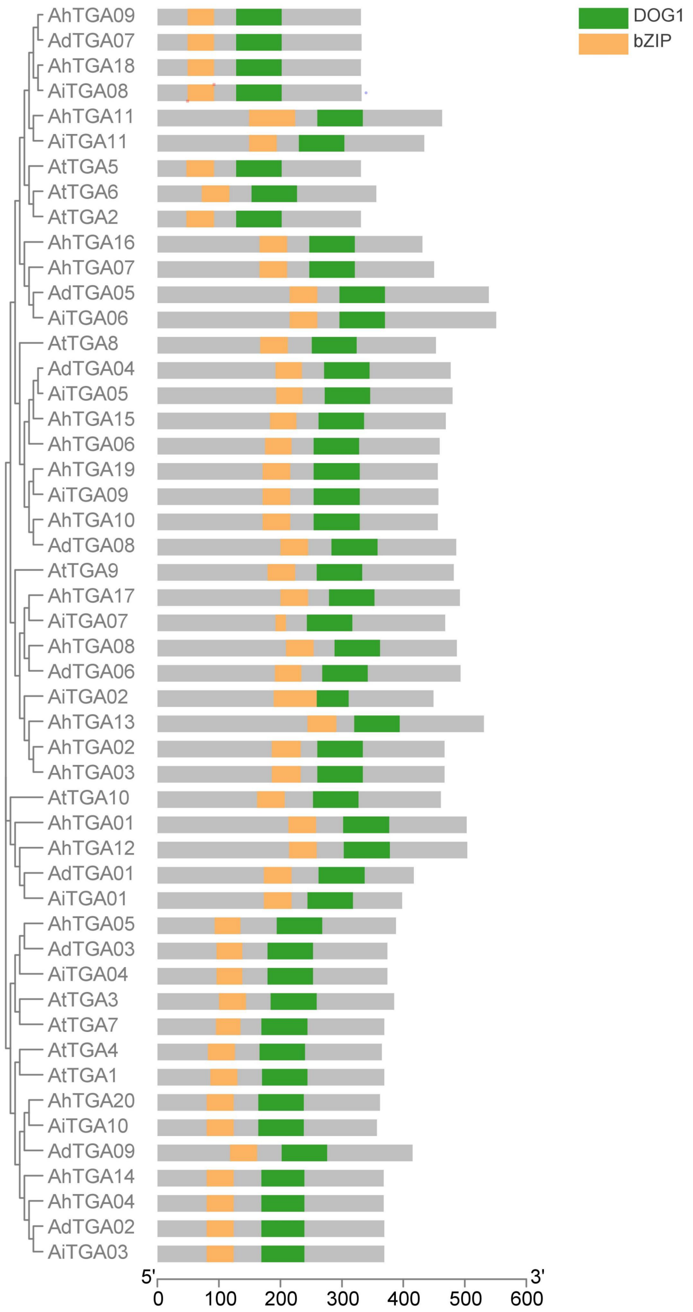

AhTGA09
AdTGA07
AhTGA18
AiTGA08
AhTGA11
AiTGA11
AtTGA5
AtTGA6
AtTGA2
AhTGA16
AhTGA07
AdTGA05
AiTGA06
AtTGA8
AdTGA04
AiTGA05
AhTGA15
AhTGA06
AhTGA19
AiTGA09
AhTGA10
AdTGA08
AtTGA9
AhTGA17
AiTGA07
AhTGA08
AdTGA06
AiTGA02
AhTGA13
AhTGA02
AhTGA03
AtTGA10
AhTGA01
AhTGA12
AdTGA01
AiTGA01
AhTGA05
AdTGA03
AiTGA04
AtTGA3
AtTGA7
AtTGA4
AtTGA1
AhTGA20
AiTGA10
AdTGA09
AhTGA14
AhTGA04
AdTGA02
AiTGA03
DOG1
bZIP
5'
3'
0
100
200
300
400
500
600

Figure S1 Comparison of conserved structural domains between *AtTGA* and peanut (cultivated peanut and wild peanuts).
