# Supplementary material for: Genome-wide analysis reveals regulatory mechanisms and expression patterns of TGA genes in peanut under abiotic stress and hormone treatments
Source: Front Plant Sci. 2023 Nov 21;14:1269200. doi: 10.3389/fpls.2023.1269200 (PMC10702600; doi:10.3389/fpls.2023.1269200)
Supplement: Supplementary file 1 [file DataSheet_1.zip › Data Sheet 1 (8)/Figure S4.pdf]

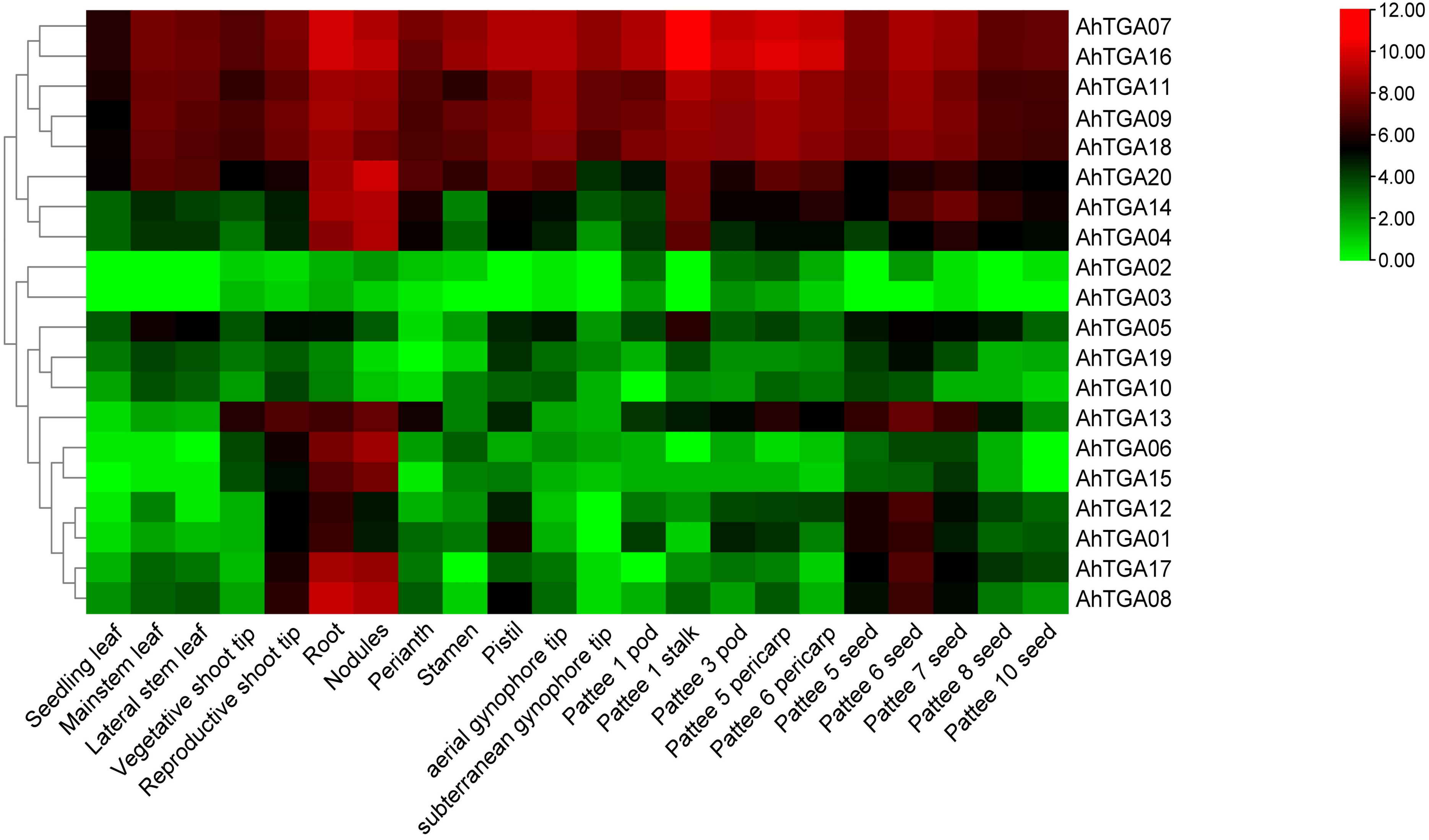

AhTGA07
AhTGA16
AhTGA11
AhTGA09
AhTGA18
AhTGA20
AhTGA14
AhTGA04
AhTGA02
AhTGA03
AhTGA05
AhTGA19
AhTGA10
AhTGA13
AhTGA06
AhTGA15
AhTGA12
AhTGA01
AhTGA17
AhTGA08
12.00
10.00
8.00
6.00
4.00
2.00
0.00
Seedling leaf
Mainstem leaf
Lateral stem leaf
Vegetative shoot tip
Reproductive shoot tip
Root
Nodules
Perianth
Stamen
Pistil
aerial gynophore tip
subterranean gynophore tip
Pattee 1 pod
Pattee 1 stalk
Pattee 3 pod
Pattee 5 pericarp
Pattee 6 pericarp
Pattee 5 seed
Pattee 6 seed
Pattee 7 seed
Pattee 8 seed
Pattee 10 seed

Figure S4 Expression profiles of 20 *AhTGAs* in 22 different tissues. Heat-map clustering based on FPKM expression of 20 *AhTGA* genes in different peanut tissues. Log2-transformed values are used in a color-coded heatmap, with bars representing normalized FPKM (Log2) expression levels, and red for higher expression , green means low.
