# Supplementary material for: Genome-wide analysis reveals regulatory mechanisms and expression patterns of TGA genes in peanut under abiotic stress and hormone treatments
Source: Front Plant Sci. 2023 Nov 21;14:1269200. doi: 10.3389/fpls.2023.1269200 (PMC10702600; doi:10.3389/fpls.2023.1269200)
Supplement: Supplementary file 1 [file DataSheet_1.zip › Data Sheet 1 (8)/Figure S5.pdf]

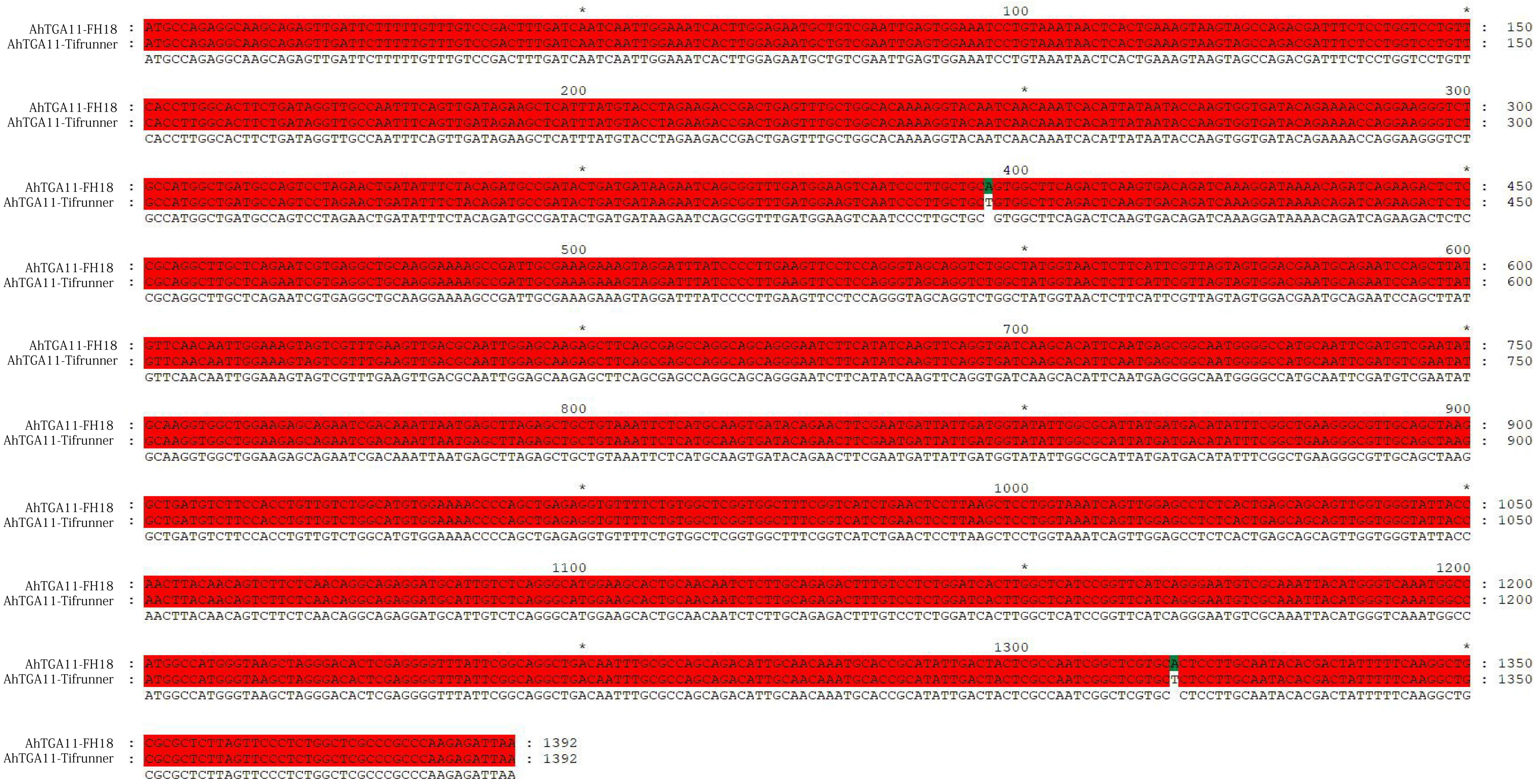
AhTGA11-FH18
AhTGA11-Tifrunner

Figure S5 The coding region gene sequence of the orthologous gene *AhTGA11*-Tifrunner (reference genome) was aligned with the alleles of *AhTGA11*-FH18.
