# Supplementary figures and images for: Genome-wide analysis reveals regulatory mechanisms and expression patterns of TGA genes in peanut under abiotic stress and hormone treatments
Source: Front Plant Sci. 2023 Nov 21;14:1269200. doi: 10.3389/fpls.2023.1269200 (PMC10702600; doi:10.3389/fpls.2023.1269200)

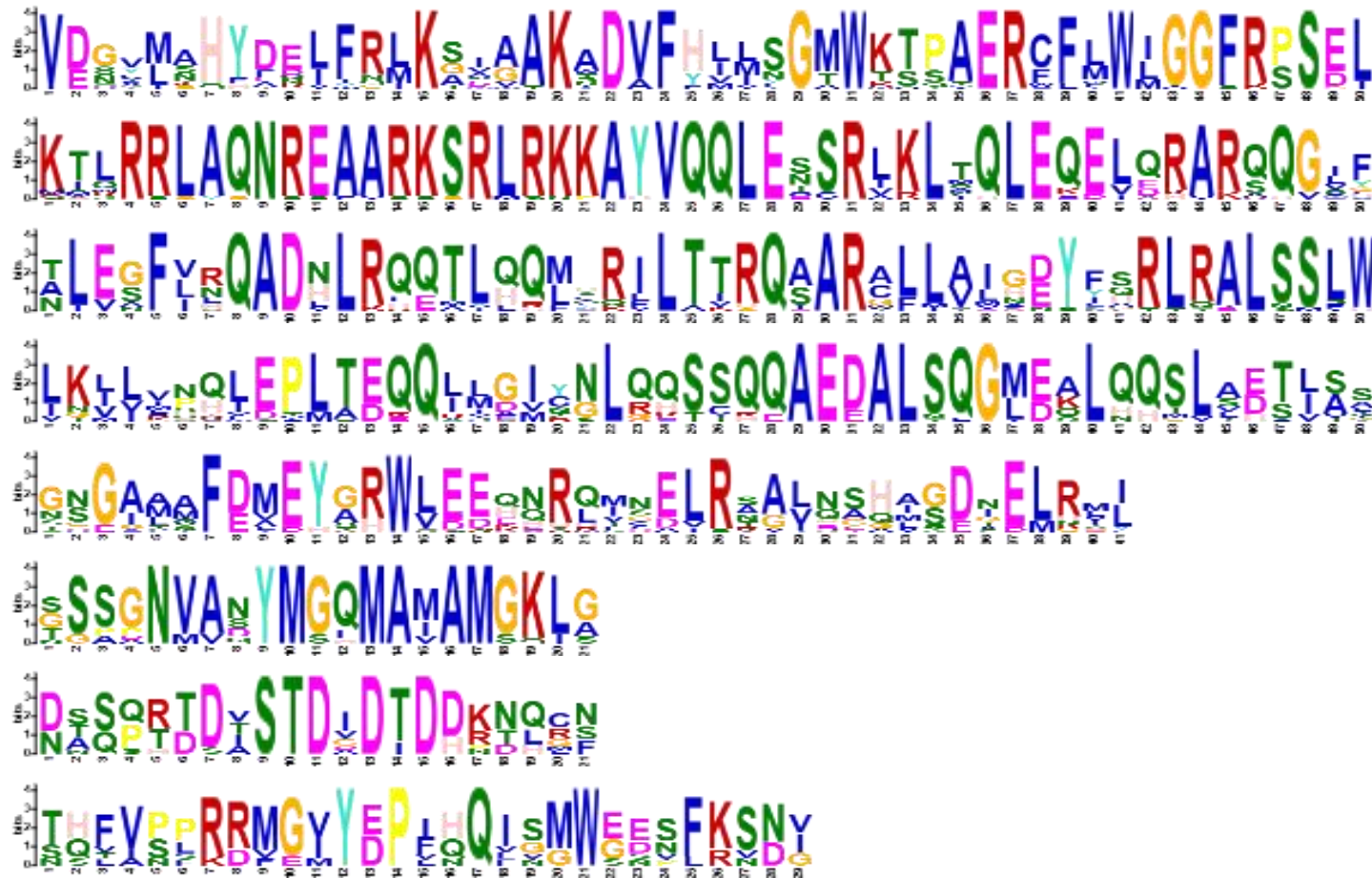

**E-value**

1.1e-1101

3.1e-1086

1.9e-910

2.7e-809

8.3e-612

6.9e-170

8.9e-082

2.2e-081

**Figure S2** Sequence logos of 10 motifs in cultivated peanut TGA proteins.

Supplement: Supplementary file 1 [file DataSheet_1.zip › Data Sheet 1 (8)/Figure S2.pdf]

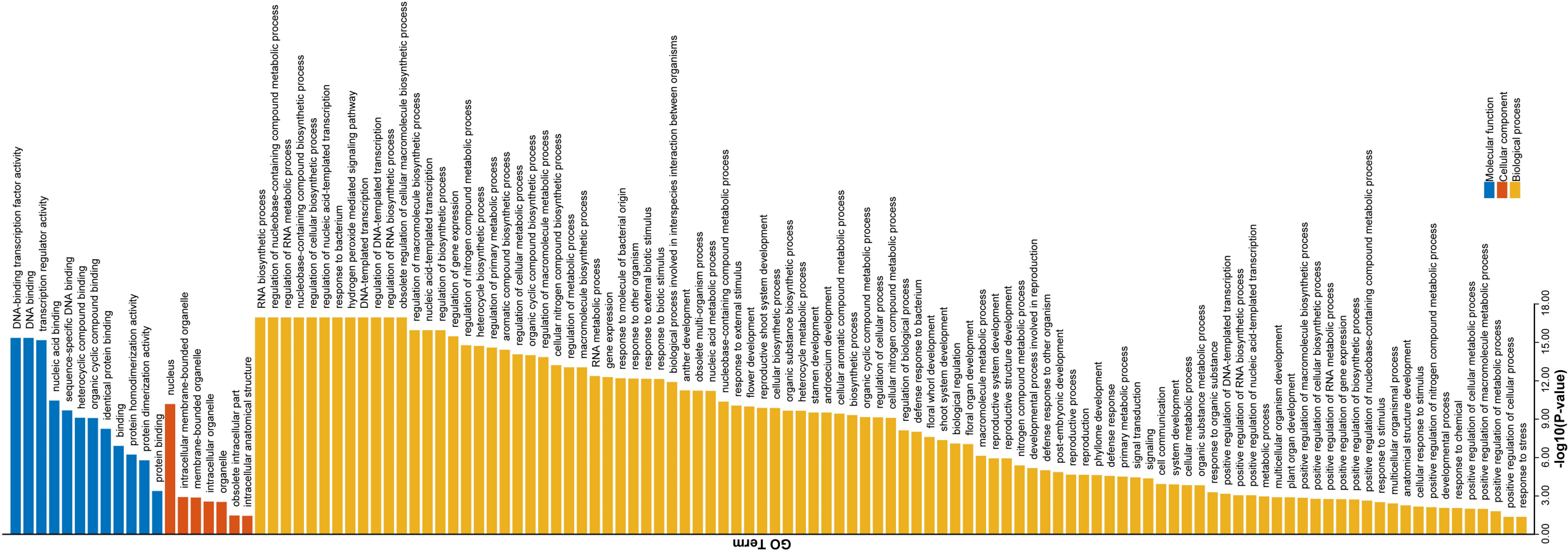

**Figure S3** Functional annotation of candidate genes interacted with *AhTGAs*.

Supplement: Supplementary file 1 [file DataSheet_1.zip › Data Sheet 1 (8)/Figure S3.pdf]
